# Diverse combination of factors associated with the development of diabetic kidney disease among data-driven diabetes subtypes: analysis of the J-DREAMS registry

**DOI:** 10.1007/s00125-025-06594-1

**Published:** 2025-11-17

**Authors:** Kiriko Watanabe-Shimoji, Hayato Tanabe, Mitsuru Ohsugi, Eiryo Kawakami, Kenichi Tanaka, Junichiro J. Kazama, Kohjiro Ueki, Michio Shimabukuro

**Affiliations:** 1 Department of Diabetes, Endocrinology, and Metabolism, Fukushima Medical University, Fukushima, Japan; 2 Department of Diabetes, Endocrinology, Metabolism, and General Internal Medicine, Fukushima Medical University, Fukushima, Japan; 3 Department of Diabetes, Endocrinology and Metabolism, Japan Institute for Health Security, Tokyo, Japan; 4 Diabetes and Metabolism Information Center, Japan Institute for Health Security, Tokyo, Japan; 5 Department of Artificial Intelligence Medicine, Graduate School of Medicine, Chiba University, Chiba, Japan; 6 Advanced Data Science Project, RIKEN Information R&D and Strategy Headquarters, RIKEN, Yokohama, Japan; 7 Department of Nephrology and Hypertension, Fukushima Medical University, Fukushima, Japan; 8 Department of Molecular Diabetic Medicine, Diabetes Research Center, Japan Institute for Health Security, Tokyo, Japan

**Keywords:** Clustering, Diabetes subtypes, Diabetic kidney disease, End-stage kidney disease, Machine learning, Type 2 diabetes

## Abstract

**Aims/hypothesis:**

Available methods for predicting the onset and progression of diabetic kidney disease (DKD) and end-stage kidney disease (ESKD) are not yet ready for clinical application. We used a Japanese diabetes cohort study (J-DREAMS) to examine whether the Ahlqvist et al diabetes clustering is useful for stratifying DKD or ESKD outcomes independent of known risk factors in real-world settings.

**Methods:**

Data-driven cluster analysis using *k*-means was performed based on GAD antibody levels, age at diagnosis, BMI, HbA_1c_ and HOMA2 estimates of beta cell function and insulin resistance in 12,093 individuals with type 1 or type 2 diabetes. The risk of developing DKD/ESKD was analysed using Kaplan–Meier analysis and the Cox proportional hazards model.

**Results:**

Diabetes clustering classified individuals in the J-DREAMS cohort into five subtypes, the clinical characteristics of which were comparable to those of the previously reported five subtypes. Kaplan–Meier curve analysis showed that events for chronic kidney disease (CKD) stages 3b, 4 and 5 were highest in the severe insulin-resistant diabetes subtype. The Cox proportional hazards model showed that the severe insulin-resistant diabetes subtype had significant HRs after correction for multiple confounding factors. The Cox proportional hazards model showed that each subtype had a diverse combination of factors associated with CKD stage 3b and proteinuria events.

**Conclusions/interpretation:**

Data-driven analysis provides diabetes subtyping, which can predict the probability of developing DKD/ESKD; each subtype has diverse combinations of factors predisposing to DKD development and progression. Data-driven diabetes subtyping to predict the likelihood of developing DKD/ESKD and mitigating predisposing factors may help personalise prevention strategies.

**Graphical Abstract:**

**Figure:**
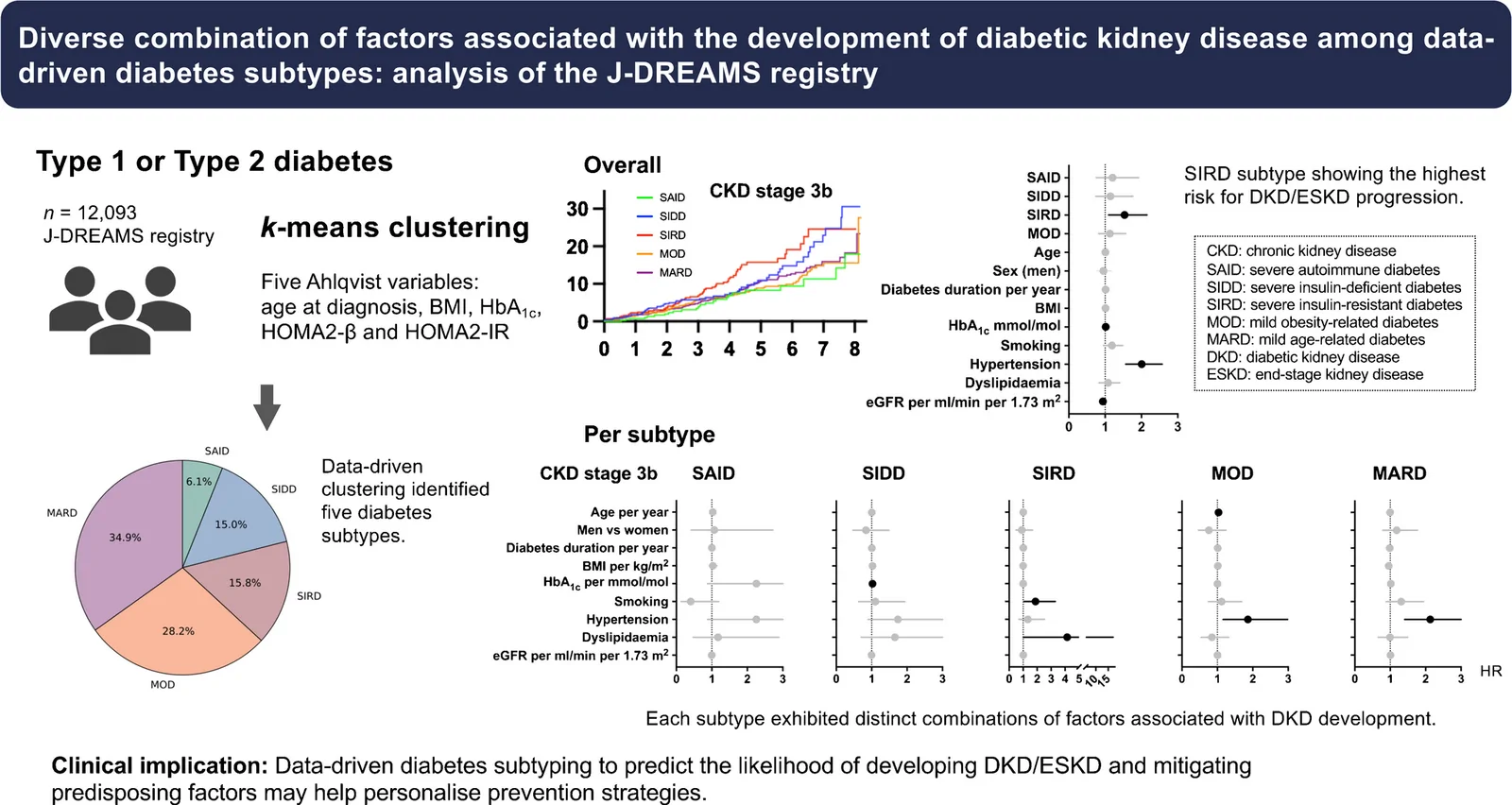

**Supplementary Information:**

The online version contains peer-reviewed but unedited supplementary material available at https://doi.org/10.1007/s00125-025-06594-1.

**Figure:**
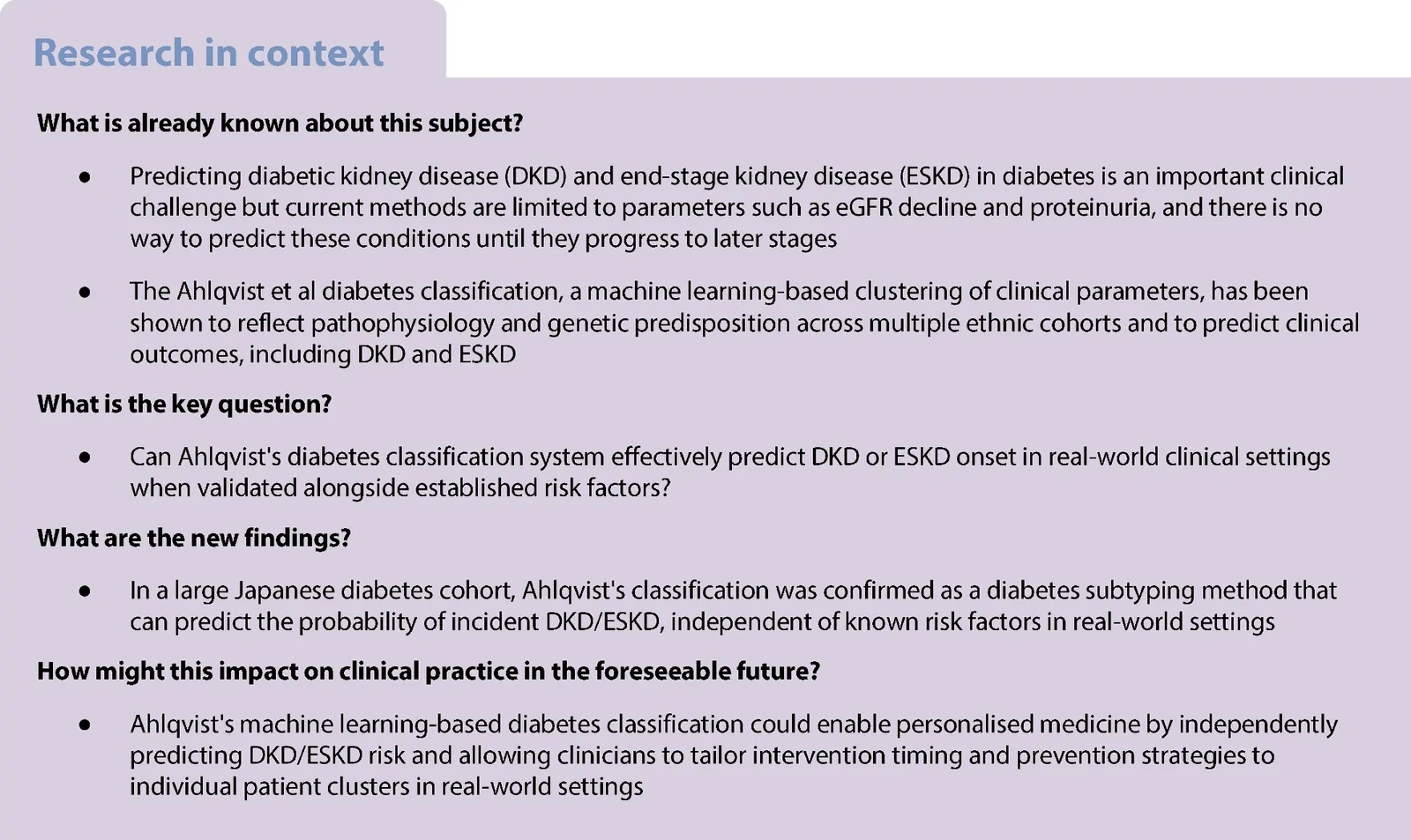

## Introduction

Diabetic kidney disease (DKD) is a common complication affecting 20–40% of individuals with diabetes, often progressing to end-stage kidney disease (ESKD) requiring dialysis or kidney transplantation. Early detection and intervention are effective in preventing progression to ESKD in individuals with diabetes [1, 2]. Regular screening for eGFR and microalbuminuria is effective for early detection in individuals with diabetes [1, 2]. However, as the process of DKD begins before the decline in eGFR and the appearance of microalbuminuria, accurately predicting the likelihood of the future development of ESKD at the time of diabetes diagnosis may allow the most appropriate preventive measures to be taken for each individual.

Several approaches have been proposed to predict the onset and progression of DKD. The first was the use of clinical predictors. Risk prediction models have been developed that combine clinical data, such as age, BMI, smoking, diabetic retinopathy, HbA_1c_, systolic blood pressure, HDL-cholesterol, triacylglycerol and urinary albumin/creatinine ratio [3]. Second was the use of biomarkers: multi-omics analysis, including genomics, metabolomics and proteomics, can be used to identify new biomarkers [4]. Third, machine learning-based clustering and precision medicine approaches have been applied. These methods offer improved predictive accuracy over traditional logistic regression models [5, 6]. Fourth, precision medicine approaches based on individual patient genetic information and metabolic profiles are expected to contribute to the diagnosis and treatment of DKD [7]. The approaches described above are expected to improve the accuracy of DKD prognosis prediction and contribute to personalised treatment; however, they are all experimental and are not yet ready for clinical application.

Using a data-driven approach, Ahlqvist et al showed that adult-onset diabetes can be classified into five subtypes that maintain their pathophysiological characteristics. The incidence of DKD and ESKD differs greatly among these subtypes [8]. This data-driven approach has identified a common diabetes subtype prone to DKD, the severe insulin-resistant diabetes (SIRD) subtype, in several ethnic groups, including Swedish [8], German [9], white [10] and Asian [11, 12]. Therefore, determining the subtype may enable effective prediction of the onset of DKD and ESKD. However, previous studies on the predictive ability of DKD using a data-driven approach did not consider other risk factors, such as age, hypertension, smoking and duration of diabetes, although they were large in scale (Ahlqvist et al, *N*=8980; Zaharia et al, *N*=1105; Dennis et al, *N*=4351) [8–10] or validated only in a single centre (Tanabe et al, *N*=1520), while multiple variables were considered [11, 12]. Therefore, whether a data-driven approach can be applied universally to real-world populations to predict DKD and ESKD is unclear [13].

In this study, we aimed to verify whether data-driven subtyping is useful for stratifying the risk of DKD and ESKD independent of other DKD risks in the Japan Diabetes compREhensive database project based on Advanced electronic Medical record System (J-DREAMS) [14], a nationwide Japanese diabetes cohort. We also aimed to clarify the combinations of factors that cause DKD in each diabetes subtype.

## Methods

### Study design and participants

The study protocol was approved by the Fukushima Medical University Ethics Committee (Number REC2022-028). We performed a retrospective cohort analysis using the J-DREAMS registry [14]. J-DREAMS is a large, nationwide database of participants with diabetes launched by the National Center for Global Health and Medicine in collaboration with the Japan Diabetes Society [15], in which cases are registered from 74 (as of June 2024) hospitals and clinics across Japan without any regional bias. Data were collected using a standard diabetes management template, which includes the diabetic disease state and comorbid conditions/complications that are collected systematically using precisely validated definitions installed on the electronic medical record (EMR) system at each facility. Participant race was determined by self-report and recorded by the attending physician within this template. The date of the first record of a participant in the database was considered the index date, and participants were followed up until the end of the available data, death or dropout from the data source, whichever was earliest. No look-back period was applied in this study, and 6 months from the index date was designated as the baseline period to identify the participants’ baseline characteristics, including DKD history.

**Fig. 1:**
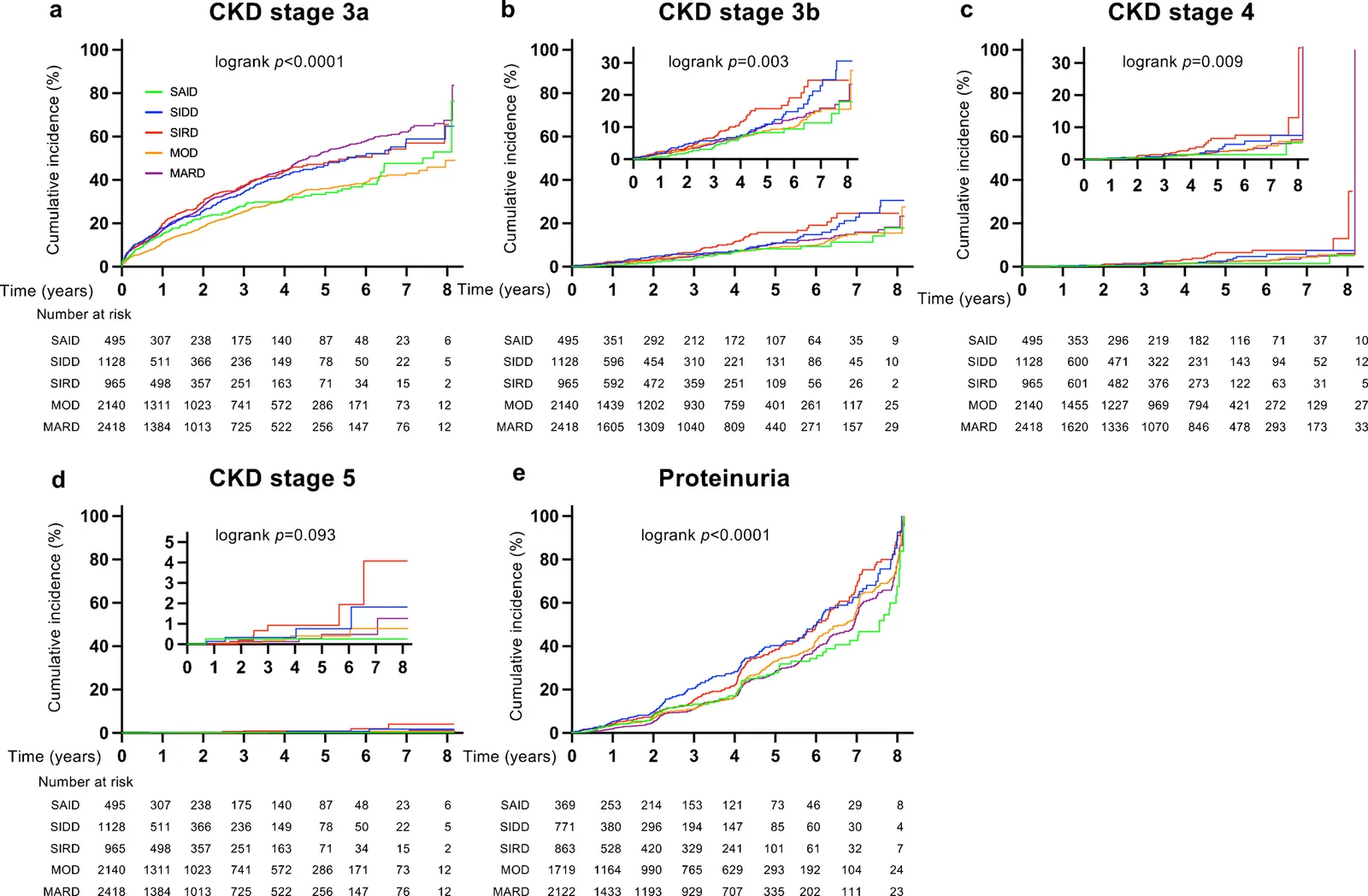
Kaplan–Meier curves for the development of (**a**) stage 3a (eGFR<60 ml/min per 1.73 m^2^), (**b**) stage 3b (eGFR<45 ml/min per 1.73 m^2^), (**c**) stage 4 (eGFR<30 ml/min per 1.73 m^2^), (**d**) stage 5 (eGFR<15 ml/min per 1.73 m^2^) and (**e**) proteinuria in individuals with SAID (green lines), SIDD (blue lines), SIRD (red lines), MOD (orange lines) and MARD (purple lines). Analyses for stages 3a, 3b, 4 and 5 were performed using Dataset B (*n*=7146) and those for proteinuria using Dataset C (*n*=5844) in a Japanese diabetes cohort database (J-DREAMS). The inset graphs show magnified views of the *y* axes to better illustrate early separation of the curves

The study population consisted of individuals with type 1 or type 2 diabetes who were being treated at a clinic or hospital and participating in J-DREAMS (electronic supplementary material [ESM] Fig. 1). The inclusion criteria were: (1) at least one visit in the database between 1 April 2015 and 31 March 2023 (index period); (2) age≥18 years at the index date; (3) diagnosis of type 1 or type 2 diabetes; and (4) Japanese race. The exclusion criteria were: (1) other forms of diabetes/secondary diabetes; (2) missing data for clustering; (3) outlier over 3 SD for BMI, HOMA2-IR or HOMA2-β; (4) fasting plasma glucose (FPG) level <3.9 mmol/l or >11.1 mmol/l. Among participants (*N*=101,155) enrolled from 1 April 2015 to 31 March 2023 in J-DREAMS, the full analysis set (Dataset A, *n*=12,093) consisted of participants after exclusion for other form/secondary diabetes (*n*=7076), diagnosis before age 18 years (*n*=1302), missing data for clustering (*n*=76,072), outlier over 3 SD (*n*=433) and FPG level <3.9 mmol/l or >11.1 mmol/l (*n*=4179). A heatmap of the missing data patterns is shown in ESM Fig. 2. Distributions of Ahlqvist’s variables, except for the GAD antibody, in J-DREAMS Dataset A (*n*=12,093) are shown in ESM Fig. 3. In Dataset A, participants were classified as having severe autoimmune diabetes (SAID) (*n*=733) if they were positive for anti-GAD antibody or diagnosed based on Japanese diagnostic criteria by their attendant diabetologists [16, 17] (ESM Fig. 1). The remaining 11,360 participants, excluding those with SAID, were subjected to *k*-means clustering (*k*=4) using the original remaining five variables (age at diagnosis, BMI, HbA_1c_, HOMA2-β and HOMA2-IR) [8]. Of Dataset A, Dataset B (*n*=7146) was extracted after the exclusion of missing eGFR data (*n*=1454); chronic kidney disease (CKD) stages G3a, G3b, G4 or G5 at baseline (*n*=3045); and no follow-up for eGFR (*n*=448); and Dataset C (*n*=5844) was extracted after exclusion of missing proteinuria data (*n*=495), proteinuria ≥ 1+ at baseline (*n*=2407) and no follow-up for proteinuria (*n*=3347). Dataset B was used to analyse DKD events (eGFR < 60, <45, <30 and <15 ml/min per 1.73 m^2^), and Dataset C was used to analyse proteinuria events. Dataset D (*n*=5230) was used for analysis of DKD events using Ahlqvist’s variables and DKD risk factors. Dataset E (*n*=3952) was extracted from Dataset A for cluster reproducibility analysis. This analysis included participants who had Ahlqvist’s variables at both the baseline and follow-up assessments (mean interval: 5.0±1.2 years).

### Definitions

Laboratory measurements including GAD antibody, HbA_1c_, serum creatinine, and lipid profiles were performed at each participating hospital using their standard laboratory protocols and commercially available assays. Given the multi-centre nature of this study involving 74 hospitals and clinics across Japan, specific reagent manufacturers and assay methods varied between institutions.

Diabetes was defined as HbA_1c _≥ 48 mmol/mol (6.5%), FPG level ≥7.0 mmol/l or current use of glucose-lowering medications [18]. Hypertension was defined as blood pressure≥140/90 mmHg or current use of antihypertensive medication(s). Dyslipidaemia was defined as a total serum cholesterol level ≥5.70 mmol/l, LDL-cholesterol level ≥3.63 mmol/l, serum triacylglycerol level ≥1.70 mmol/l, a serum HDL-cholesterol level of <1.04 mmol/l or current use of lipid-lowering medications. Smoking status was defined as the participant being a past or current smoker, and non-smoking status was defined as a participant who had never smoked. Renal function was assessed using the Japanese formula for GFR estimation: eGFR (ml/min per 1.73 m^2^) =194 × serum creatinine (mg/dl)^−1.094^ × age (years)^−0.287^ [19]. Dipstick urinalysis was performed to detect proteinuria using spontaneously voided fresh urine, which was analysed within minutes of collection. In this study, proteinuria was defined as 1+ or greater. Developing DKD was defined as the development of eGFR<60 ml/min per 1.73 m^2^ or proteinuria. DKD was subclassified into stage 3a (eGFR<60 ml/min per 1.73 m^2^), stage 3b (<45), stage 4 (<30) and stage 5 (<15) according to CKD staging [1].

### Statistical analysis

Continuous and parametric variables were expressed as mean ± SD, while non-parametric variables were expressed as median (IQR). Outliers for BMI, HOMA2-IR and HOMA2-β were determined using the Kolmogorov–Smirnov test. Between-group differences were analysed using one-way ANOVA or the Kruskal–Wallis test. Categorical variables were expressed as percentages, and intergroup differences were analysed using the χ^2^ test.

The *k*-means clustering was performed using the fpc package and the kmeansruns function (run 2000 times) in R. The appropriate number of clusters was estimated using the elbow method [20]. Clustering was performed using five continuous variables (age at onset, BMI, HbA_1c_, HOMA2-β, HOMA2-IR) and one binary variable (GAD antibody) in accordance with Ahlqvist’s method [8, 12]. Individuals who were GAD antibody-positive or diagnosed with type 1 diabetes based on Japanese diagnostic criteria [16, 17] were manually assigned to the SAID cluster. To avoid stratification by sex, male and female participants were clustered separately. The stability of the clustering was evaluated using the Jaccard index after resampling the dataset 2000 times [21]. For the cluster reproducibility analysis of Dataset E, we clustered the participants twice at baseline and during follow-up to assess migration patterns between clusters.

The Kaplan–Meier curve analysis with logrank tests for between-group comparisons was performed to estimate cumulative incidence rates.

Using unadjusted and multivariable-adjusted Cox proportional hazards models, we evaluated the risk of eGFR events (CKD stage 3a: eGFR<60 ml/min per 1.73 m^2^, stage 3b: eGFR<45, stage 4: eGFR<30 and stage 5: eGFR<15) and proteinuria events, calculating HRs and 95% CIs. We constructed five multivariable-adjusted Cox proportional hazards models as follows: Model 1 included basic covariates: each subtype vs the mild age-related diabetes (MARD) subtype, age (years), male vs female, and diabetes duration (years). Model 5 was the fully adjusted model, which added known DKD risk factors to Model 1: BMI (kg/m^2^), HbA_1c_ (mmol/mol), current or past smoking vs no smoking history, hypertension present vs absent, dyslipidaemia present vs absent, and baseline eGFR (ml/min per 1.73 m^2^). To assess the impact of key variables used for clustering, we also constructed Model 2 (based on Model 5, but removing both BMI and HbA_1c_), Model 3 (based on Model 5, but removing only BMI), and Model 4 (based on Model 5, but removing only HbA_1c_).

As a sensitivity analysis, we examined whether clustering with the Ahlqvist variables plus DKD risk factors outperformed Ahlqvist's subtypes in predicting DKD risk. Ahlqvist’s five variables (age at diagnosis, BMI, HbA_1c_, HOMA2-β, HOMA2-IR) plus five DKD risk factors (eGFR, systolic blood pressure, HDL-cholesterol, LDL-cholesterol, triacylglycerol) were used for *k*-means clustering. In determining the optimal number of clusters, the Elbow method indicated four clusters for type 2 diabetes, and ultimately five clusters were adopted for individuals with type 1 or type 2 diabetes. Then, comparisons using Kaplan–Meier curves and Cox regression analysis were made using these five clusters as noted above.

Per subtype, a multivariate model was created using the same combinations of variables as in overall Model 5 for stage 3b and proteinuria events. Analysis for CKD stages 4 and 5 was not performed due to the small number of cases. To verify the effects of subtype-specific risk factors observed in the stratified analysis, we evaluated the interaction terms between diabetes subtypes and major DKD risk factors (age, sex, diabetes duration, BMI, HbA_1c_, hypertension, dyslipidaemia, smoking) in Cox proportional hazards models using likelihood ratio tests.

Assumptions of the Cox proportional hazards model were tested using the Schoenfeld residuals test. The Grambsch–Therneau test was used to perform global testing of each model and each covariate. If a violation of the proportional hazards assumption was detected (*p*<0.05), we confirmed this visually (log–log survival curves, scaled Schoenfeld residual plots) and performed sensitivity analyses using stratified Cox regression models as necessary. Assessment of multicollinearity variance inflation factor (VIF) analysis was performed for the covariates in Model 5 of Table 1.

**Table 1:** Overall Cox proportional hazards model for eGFR and proteinuria events

Variable	Events (%)	Censored	Model 1			Model 2			Model 3			Model 4			Model 5			
			HR (95% CI)	*p* value	*q* value	HR (95% CI)	*p* value	*q* value	HR (95% CI)	*p* value	*q* value	HR (95% CI)	*p* value	*q* value	HR (95% CI)	*p* value	*q* value	VIF
CKD stage 3a																		
SAID	128 (25.9)	367	1.30 (1.07, 1.58)	0.009	0.045	1.27 (1.10, 1.48)	0.018	0.090	1.14 (0.93, 1.40)	0.205	0.280	1.27 (1.04, 1.55)	0.017	0.029	1.14 (0.93, 1.40)	0.203	0.489	1.401
SIDD	269 (23.8)	859	1.45 (1.24, 1.68)	<0.001	0.002	1.70 (1.46, 1.98)	<0.001	0.003	1.23 (1.01, 1.50)	0.035	0.060	1.69 (1.52, 1.97)	<0.001	0.001	1.23 (1.01, 1.50)	0.036	0.138	2.487
SIRD	277 (28.7)	688	1.55 (1.34, 1.79)	<0.001	0.001	1.28 (1.10, 1.48)	0.001	0.005	1.29 (1.11, 1.49)	0.001	0.002	1.26 (1.08, 1.50)	0.003	0.007	1.28 (1.10, 1.50)	0.002	0.012	1.657
MOD	476 (22.2)	1664	1.09 (0.94, 1.26)	0.269	0.448	1.05 (0.91, 1.22)	0.487	0.609	1.05 (0.90, 1.21)	0.543	0.592	1.05 (0.90, 1.21)	0.548	0.613	1.04 (0.90, 1.21)	0.562	0.777	2.373
MARD	801 (33.1)	1617	1.00 (ref)	–	–	1.00 (ref)	–		1.00 (ref)	–		1.00 (ref)	–		1.00 (ref)	–	–	–
Age per year			1.05 (1.04, 1.05)	<0.001	0.002	1.02 (1.02, 1.03)	<0.001	0.001	1.02 (1.01, 1.03)	<0.001	0.002	1.02 (1.02, 1.03)	<0.001	0.003	1.02 (1.01, 1.03)	<0.001	0.055	2.709
Men vs women			1.11 (1.01, 1.22)	0.031	0.039	0.95 (0.85, 1.05)	0.314	0.785	0.93 (0.84, 1.04)	0.235	0.282	0.95 (0.85, 1.05)	0.320	0.427	0.94 (0.84, 1.04)	0.237	0.513	1.305
Diabetes duration per year			1.00 (0.99, 1.00)	0.275	0.458	1.00 (0.99, 1.00)	0.238	0.595	1.00 (0.99, 1.00)	0.210	0.280	1.00 (0.99, 1.00)	0.275	0.413	1.00 (0.99, 1.00)	0.222	0.498	1.662
BMI per kg/m^2^						–	–		–	–		1.00 (0.99, 1.01)	0.617	0.617	1.00 (0.99, 1.01)	0.902	0.989	1.427
HbA_1c_ per mmol/mol						–	–		1.01 (1.01, 1.02)	<0.001	0.002	–	–		1.02 (1.01, 1.02)	<0.001	0.007	2.075
Current or ex-smoking vs never smoking						1.13 (1.02, 1.25)	0.016	0.080	1.13 (1.02, 1.25)	0.017	0.034	1.13 (1.02, 1.26)	0.015	0.029	1.13 (1.02, 1.25)	0.017	0.074	1.218
Hypertension yes vs no						1.38 (1.25, 1.51)	<0.001	0.002	1.38 (1.25, 1.53)	<0.001	0.002	1.37 (1.24, 1.52)	<0.001	0.003	1.38 (1.25, 1.53)	<0.001	0.805	1.198
Dyslipidaemia yes vs no						1.04 (0.93, 1.16)	0.524	0.715	1.03 (0.92, 1.15)	0.621	0.621	1.03 (0.92, 1.16)	0.562	0.613	1.03 (0.92, 1.15)	0.632	0.007	1.074
eGFR per ml/min per 1.73 m^2^						0.91 (0.91, 0.92)	<0.001	<0.001	0.91 (0.91, 0.92)	<0.001	0.002	0.91 (0.91, 0.92)	<0.001	0.003	0.91 (0.91, 0.92)	<0.001	0.007	1.372
CKD stage 3b																		
SAID	24 (4.8)	471	0.96 (0.70, 1.32)	0.814	0.861	1.32 (0.83, 2.08)	0.241	0.560	1.18 (0.74, 1.89)	0.471	0.572	1.33 (0.84, 2.12)	0.220	0.345	1.20 (0.75, 1.92)	0.441	0.735	
SIDD	59 (5.2)	1069	1.46 (1.19, 1.79)	<0.001	0.002	1.68 (1.20, 2.36)	0.003	0.005	1.15 (0.75, 1.77)	0.524	0.572	1.65 (1.17, 2.32)	0.004	0.016	1.14 (0.74, 1.76)	0.543	0.777	
SIRD	67 (6.9)	898	1.89 (1.59, 2.26)	<0.001	0.001	1.60 (1.17, 2.20)	0.004	0.007	1.61 (1.17, 2.21)	0.003	0.012	1.51 (1.08, 2.11)	0.017	0.051	1.53 (1.10, 2.15)	0.013	0.060	
MOD	114 (5.3)	2026	1.29 (1.07, 1.56)	0.009	0.033	1.16 (0.84, 1.60)	0.364	0.609	1.15 (0.83, 1.59)	0.383	0.572	1.13 (0.82, 1.56)	0.465	0.536	1.13 (0.82, 1.56)	0.459	0.745	
MARD	136 (5.6)	2282	1.00 (ref)	–	–	1.00 (ref)	–	–	1.00 (ref)	–		1.00 (ref)	–		1.00 (ref)	–	–	
Age per year			1.05 (1.04, 1.06)	<0.001	0.002	1.01 (0.99, 1.02)	0.312	0.520	1.01 (0.99, 1.02)	0.350	0.572	1.01 (1.00, 1.02)	0.230	0.345	1.01 (0.99, 1.02)	0.279	0.575	
Men vs women			1.19 (1.05, 1.35)	0.007	0.027	0.96 (0.76, 1.22)	0.740	0.925	0.95 (0.75, 1.20)	0.672	0.672	0.96 (0.76, 1.22)	0.749	0.749	1.01 (1.00, 1.02)	0.681	0.835	
Diabetes duration per year			1.01 (1.00, 1.02)	0.028	0.070	1.01 (1.00, 1.02)	0.105	0.525	1.01 (1.00, 1.02)	0.112	0.269	1.01 (1.00, 1.02)	0.076	0.182	0.95 (0.75, 1.20)	0.090	0.269	
BMI per kg/m^2^						–	–	–	–	–		1.01 (0.99, 1.04)	0.290	0.387	1.01 (0.99, 1.03)	0.420	0.718	
HbA_1c_ per mmol/mol						–	–	–	1.02 (1.00, 1.03)	0.004	0.012	–	–		1.02 (1.00, 1.03)	0.006	0.032	
Current or ex-smoking vs never smoking						1.19 (0.96, 1.49)	0.116	0.290	1.18 (0.95, 1.47)	0.142	0.284	1.20 (0.96, 1.50)	0.103	0.206	1.19 (0.96, 1.48)	0.129	0.349	
Hypertension yes vs no						2.03 (1.59, 2.59)	<0.001	<0.001	2.04 (1.60, 2.60)	<0.001	0.006	1.97 (1.55, 2.54)	<0.001	0.006	2.01 (1.57, 2.57)	<0.001	0.007	
Dyslipidaemia yes vs no						1.11 (0.86, 1.44)	0.425	0.715	1.09 (0.84, 1.41)	0.501	0.572	1.10 (0.84, 1.42)	0.491	0.536	1.08 (0.83, 1.40)	0.556	0.777	
eGFR per ml/min per 1.73 m^2^						0.94 (0.93, 0.95)	<0.001	<0.001	0.94 (0.93, 0.95)	<0.001	0.006	0.94 (0.93, 0.95)	<0.001	0.006	0.94 (0.93, 0.95)	<0.001	0.007	
CKD stage 4																		
SAID	5 (1.0)	490	1.11 (0.70, 1.76)	0.667	0.861	0.99 (0.36, 2.67)	0.978	0.978	0.93 (0.34, 2.53)	0.883	0.939	1.01 (0.37, 2.74)	0.981	0.981	0.95 (0.35, 2.61)	0.928	0.989	
SIDD	14 (1.2)	1114	1.36 (0.99, 1.88)	0.061	0.076	1.89 (0.94, 3.88)	0.074	0.093	1.41 (0.58, 3.44)	0.450	0.894	1.82 (0.90, 3.67)	0.095	0.285	1.39 (0.57, 3.39)	0.470	0.745	
SIRD	22 (2.3)	943	2.31 (1.79, 2.98)	<0.001	0.001	2.32 (1.27, 4.22)	0.006	0.008	2.33 (1.28, 4.24)	0.006	0.036	2.11 (1.11, 3.99)	0.023	0.138	2.13 (1.12, 4.05)	0.021	0.085	
MOD	28 (1.3)	2112	1.13 (0.84, 1.51)	0.425	0.531	1.27 (0.67, 2.41)	0.467	0.609	1.26 (0.67, 2.40)	0.473	0.894	1.22 (0.64, 2.32)	0.542	0.846	1.22 (0.64, 2.33)	0.539	0.777	
MARD	32 (1.3)	2386	1.00 (ref)	–	–	1.00 (ref)	–	–	1.00 (ref)	–	–	1.00 (ref)	–	–	1.00 (ref)	–	–	
Age per year			1.03 (1.02, 1.04)	<0.001	0.002	1.00 (0.98, 1.03)	0.921	0.921	1.00 (0.98, 1.03)	0.939	0.939	1.00 (0.98, 1.03)	0.773	0.919	1.00 (0.98, 1.03)	0.798	0.924	
Men vs women			1.28 (1.05, 1.56)	0.016	0.027	1.15 (0.71, 1.85)	0.570	0.925	1.14 (0.71, 1.83)	0.596	0.894	1.15 (0.72, 1.85)	0.564	0.846	1.14 (0.71, 1.83)	0.589	0.781	
Diabetes duration per year			1.02 (1.01, 1.03)	0.003	0.015	1.00 (0.97, 1.02)	0.748	0.935	1.00 (0.97, 1.02)	0.727	0.918	1.00 (0.97, 1.02)	0.842	0.919	1.00 (0.97, 1.02)	0.810	0.924	
BMI per kg/m^2^						–	–	–	–	–	–	1.02 (0.98, 1.07)	0.372	0.846	1.02 (0.97, 1.07)	0.419	0.718	
HbA_1c_ per mmol/mol						–	–	–	1.01 (0.99, 1.03)	0.296	0.888	–	–	–	1.01 (0.99, 1.03)	0.330	0.631	
Current or ex-smoking vs never smoking						1.08 (0.70, 1.67)	0.729	0.729	1.07 (0.69, 1.65)	0.765	0.918	1.09 (0.71, 1.69)	0.689	0.919	1.08 (0.70, 1.68)	0.724	0.856	
Hypertension yes vs no						1.64 (1.04, 2.59)	0.035	0.044	1.65 (1.04, 2.61)	0.033	0.132	1.58 (0.99, 2.52)	0.056	0.224	1.59 (1.00, 2.54)	0.052	0.188	
Dyslipidaemia yes vs no						0.87 (0.53, 1.41)	0.566	0.715	0.86 (0.53, 1.40)	0.540	0.894	0.85 (0.52, 1.38)	0.516	0.846	0.84 (0.52, 1.38)	0.496	0.768	
eGFR per ml/min per 1.73 m^2^						0.95 (0.93, 0.96)	<0.001	<0.001	0.95 (0.93, 0.96)	<0.001	0.012	0.95 (0.93, 0.96)	<0.001	0.012	0.95 (0.93, 0.96)	<0.001	0.007	
CKD stage 5																		
SAID	1 (0.2)	495	0.82 (0.08, 7.96)	0.861	0.861	0.89 (0.09, 8.76)	0.917	0.978	0.92 (0.07, 7.39)	0.782	0.999	0.83 (0.08, 8.28)	0.872	0.984	0.64 (0.06, 6.80)	0.711	0.856	
SIDD	4 (0.4)	1124	2.24 (0.52, 9.67)	0.281	0.281	2.22 (0.50, 9.77)	0.292	0.292	1.03 (0.15, 6.87)	0.978	0.999	2.36 (0.53, 10.45)	0.257	0.771	1.04 (0.15, 7.00)	0.968	0.989	
SIRD	6 (0.6)	959	3.34 (0.90, 12.4)	0.071	0.071	2.62 (0.69, 9.97)	0.158	0.158	2.68 (0.71, 10.20)	0.147	0.724	3.15 (0.78, 12.67)	0.107	0.642	3.31 (0.82, 13.3)	0.091	0.269	
MOD	5 (0.2)	2135	0.94 (0.21, 4.16)	0.937	0.937	0.90 (0.20, 4.01)	0.890	0.890	0.90 (0.20, 4.05)	0.894	0.999	0.95 (0.21, 4.28)	0.946	0.984	0.95 (0.21, 4.32)	0.949	0.989	
MARD	5 (0.2)	2413	1.00 (ref)	–	–	1.00 (ref)	–	–	1.00 (ref)	–	–	1.00 (ref)	–	–	1.00 (ref)	–	–	
Age per year			0.99 (0.95, 1.04)	0.743	0.743	0.97 (0.93, 1.02)	0.299	0.520	0.97 (0.93, 1.02)	0.307	0.921	0.97 (0.92, 1.02)	0.223	0.771	0,97 (0.92, 1.02)	0.213	0.494	
Men vs women			1.29 (0.51, 3.28)	0.588	0.588	1.03 (0.37, 2.88)	0.953	0.953	1.00 (0.36, 2.77)	0.999	0.999	1.04 (0.37, 2.93)	0.938	0.984	1.01 (0.36, 2.82)	0.986	0.989	
Diabetes duration per year			1.00 (0.95, 1.07)	0.902	0.919	1.00 (0.94, 1.06)	0.945	0.945	1.00 (0.94, 1.06)	0.953	0.999	1.00 (0.94, 1.06)	0.984	0.984	1.00 (0.94, 1.06)	0.983	0.989	
BMI per kg/m^2^						–	–	–	–	–	–	0.96 (0.86, 1.06)	0.424	0.797	0.95 (0.86, 1.06)	0.361	0.670	
HbA_1c_ per mmol/mol						–	–	–	1.03 (0.99, 1.07)	0.181	0.724	–	–	–	1.03 (0.99, 1.08)	0.154	0.385	
Current or ex-smoking vs never smoking						1.50 (0.56, 3.63)	0.420	0.525	1.34 (0.52, 3.50)	0.544	0.999	1.36 (0.52, 3.56)	0.531	0.797	1.30 (0.50, 3.41)	0.588	0.781	
Hypertension yes vs no						1.39 (0.54, 4.03)	0.499	0.499	1.50 (0.56, 4.03)	0.424	0.999	1.64 (0.60, 4.50)	0.338	0.797	1.66 (0.60, 4.60)	0.327	0.631	
Dyslipidaemia yes vs no						1.44 (0.41, 5.09)	0.572	0.715	1.41 (0.40, 4.97)	0.598	0.999	1.52 (0.42, 5.44)	0.519	0.797	1.50 (0.42, 5.37)	0.535	0.777	
eGFR per ml/min per 1.73 m^2^						0.97 (0.93, 1.00)	0.066	0.066	0.97 (0.93, 1.00)	0.056	0.672	0.96 (0.93, 1.00)	0.066	0.642	0.97 (0.93, 1.00)	0.055	0.188	
Proteinuria																		
SAID	74 (20.3)	295	1.12 (0.86, 1.45)	0.413	0.861	1.14 (0.87, 1.49)	0.336	0.560	1.01 (0.77, 1.33)	0.928	0.004	1.17 (0.90, 1.54)	0.229	0.344	1.05 (0.81, 1.39)	0.678	0.835	1.350
SIDD	149 (19.3)	622	1.62 (1.32, 1.99)	<0.001	0.002	1.63 (1.33, 2.00)	<0.001	0.001	1.15 (0.89, 1.50)	0.285	0.928	1.53 (1.24, 1.88)	<0.001	0.004	1.13 (0.87, 1.46)	0.371	0.670	2.272
SIRD	193 (22.4)	670	1.45 (1.21, 1.74)	<0.001	0.001	1.32 (1.10, 1.58)	0.003	0.007	1.33 (1.10, 1.60)	0.002	0.444	1.15 (0.95, 1.40)	0.150	0.274	1.18 (0.97, 1.43)	0.103	0.291	1.556
MOD	389 (22.6)	1330	1.26 (1.05, 1.50)	0.013	0.033	1.22 (1.02, 1.46)	0.031	0.155	1.21 (1.01, 1.45)	0.035	0.006	1.13 (0.95, 1.37)	0.160	0.274	1.14 (0.95, 1.37)	0.152	0.385	2.235
MARD	405 (19.1)	1717	1.00 (ref)	–	–	1.00 (ref)	–	–	1.00 (ref)	–	–	1.00 (ref)	–		1.00 (ref)	–	–	
Age per year			1.00 (1.00, 1.01)	0.187	0.234	1.00 (0.99, 1.00)	0.477	0.596	1.00 (0.99, 1.00)	0.389	0.084	1.00 (0.99, 1.01)	0.922	0.930	1.00 (0.99, 1.01)	0.950	0.989	2.661
Men vs women			1.16 (1.03, 1.31)	0.014	0.027	1.13 (0.99, 1.29)	0.075	0.375	1.11 (0.97, 1.26)	0.142	0.519	1.16 (1.01, 1.31)	0.033	0.079	1.13 (0.99, 1.29)	0.067	0.218	1.407
Diabetes duration per year			1.00 (0.99, 1.01)	0.919	0.919	1.00 (0.99, 1.01)	0.634	0.935	1.00 (0.99, 1.01)	0.656	0.284	1.00 (0.99, 1.01)	0.930	0.930	1.00 (0.99, 1.010	0.963	0.989	1.699
BMI per kg/m^2^						–	–	–	–	–	–	1.03 (1.02, 1.04)	<0.001	0.004	1.03 (1.01, 1.04)	<0.001	0.007	1.371
HbA_1c_ per mmol/mol						–	–	–	1.01 (1.01, 1.02)	<0.001	0.787	–	–	–	1.01 (1.01, 1.02)	<0.001	0.007	1.976
Current or ex-smoking vs never smoking						1.06 (0.94, 1.21)	0.352	0.525	1.07 (0.94, 1.22)	0.296	0.004	1.06 (0.94, 1.21)	0.329	0.439	1.07 (0.94, 1.22)	0.283	0.575	1.331
Hypertension yes vs no						1.29 (1.14, 1.47)	<0.001	<0.001	1.29 (1.13, 1.46)	<0.001	0.444	1.22 (1.08, 1.39)	0.002	0.006	1.23 (1.08, 1.40)	0.002	0.012	1.202
Dyslipidaemia yes vs no						1.00 (0.87, 1.16)	0.993	0.993	0.99 (0.85, 1.13)	0.839	0.004	0.97 (0.84, 1.13)	0.717	0.860	0.96 (0.83, 1.11)	0.608	0.790	1.060
eGFR per ml/min per 1.73 m^2^						0.99 (0.99, 1.00)	<0.001	<0.001	0.99 (0.99, 1.00)	<0.001	0.444	0.99 (0.99, 1.00)	<0.001	0.004	0.99 (0.99, 1.00)	<0.001	0.007	1.486

–, not applicable or not included in the model; Ref, reference

The statistical significance level was set at *p*<0.05 unless otherwise specified. Statistical analyses were performed using IBM SPSS Statistics for Windows, version 29.0 (IBM, Armonk, NY, USA) and R, version 4.3.3 (R Foundation for Statistical Computing, Vienna, Austria; https://www.R-project.org/).

## Results

### Baseline characteristics of the study participants

Clustering was performed on the J-DREAMS database for 12,093 individuals (Dataset A) (ESM Fig. 1). A group of 733 (6.1%) individuals was determined to have SAID based on the definition of GAD antibody positivity or type 1 diabetes. For the remaining 11,360 individuals, the elbow method determined that *k*=4 was the optimal number of clusters for male and female participants. The *k*-means method was used to separate these four clusters (ESM Fig. 4). Pie charts of the five subtypes are illustrated in ESM Fig. 5a with those of previous reports [8, 11], showing SAID (*n*=733, 6.1%), severe insulin-deficient diabetes (SIDD) (*n*=1,814, 15.0%), SIRD (*n*=1,914, 15.8%), mild obesity-related diabetes (MOD) (*n*=3,413, 28.2%) and MARD (*n*=4,219, 34.9%). In clinical characteristics of diabetes subtypes (ESM Fig. 5b), SAID was characterised by a young age of onset (46±15 years) and the highest rate of insulin treatment (79.0%). Individuals with SIDD had the highest HbA_1c_ level (61.4±16.5 mmol/mol, 9.6±1.2%) and the lowest HOMA2-β (43.7±25.0). Those with SIRD had the highest BMI (29.2±6.8 kg/m^2^) and HOMA2-IR (4.88±2.03). MOD was characterised by a relatively young age at onset (40±8 years) and moderate obesity (BMI 26.6±5.5 kg/m^2^). MARD was characterised by the oldest age at onset (61±8 years) and relatively mild metabolic abnormalities.

Among two other analysis sets of Dataset B (*n*=7146) and Dataset C (*n*=5844) (ESM Fig. 1), the distribution of cluster types (SAID: 6.9% and 6.3%, SIDD: 15.8% and 13.2%, SIRD: 13.5% and 14.8%, MOD: 29.1% and 29.4%, and MARD: 33.8% and 36.3% in Dataset B and Dataset C, respectively) and clinical characteristics were similar to those of Dataset A (ESM Tables 1, 2).

### Kaplan–Meier curves for eGFR and proteinuria events

As shown in the Kaplan–Meier curve analysis (Fig. 1a–d), the cumulative events for CKD stage 3a were as follows: MARD>SIDD=SIRD, SAID and MOD. The cumulative events for CKD stage 3b were as follows: SIRD>SIDD>SAID=MOD=MARD. The cumulative events of CKD stages 4 and 5 were the highest in the SIRD subtype. Kaplan–Meier curve analysis (Dataset C, *n*=5844) showed that the cumulative events for proteinuria were SIRD>SIDD=MOD=MARD>SAID (Fig. 1e).

### Cox proportional hazards model in the overall analysis

**Fig. 2:**
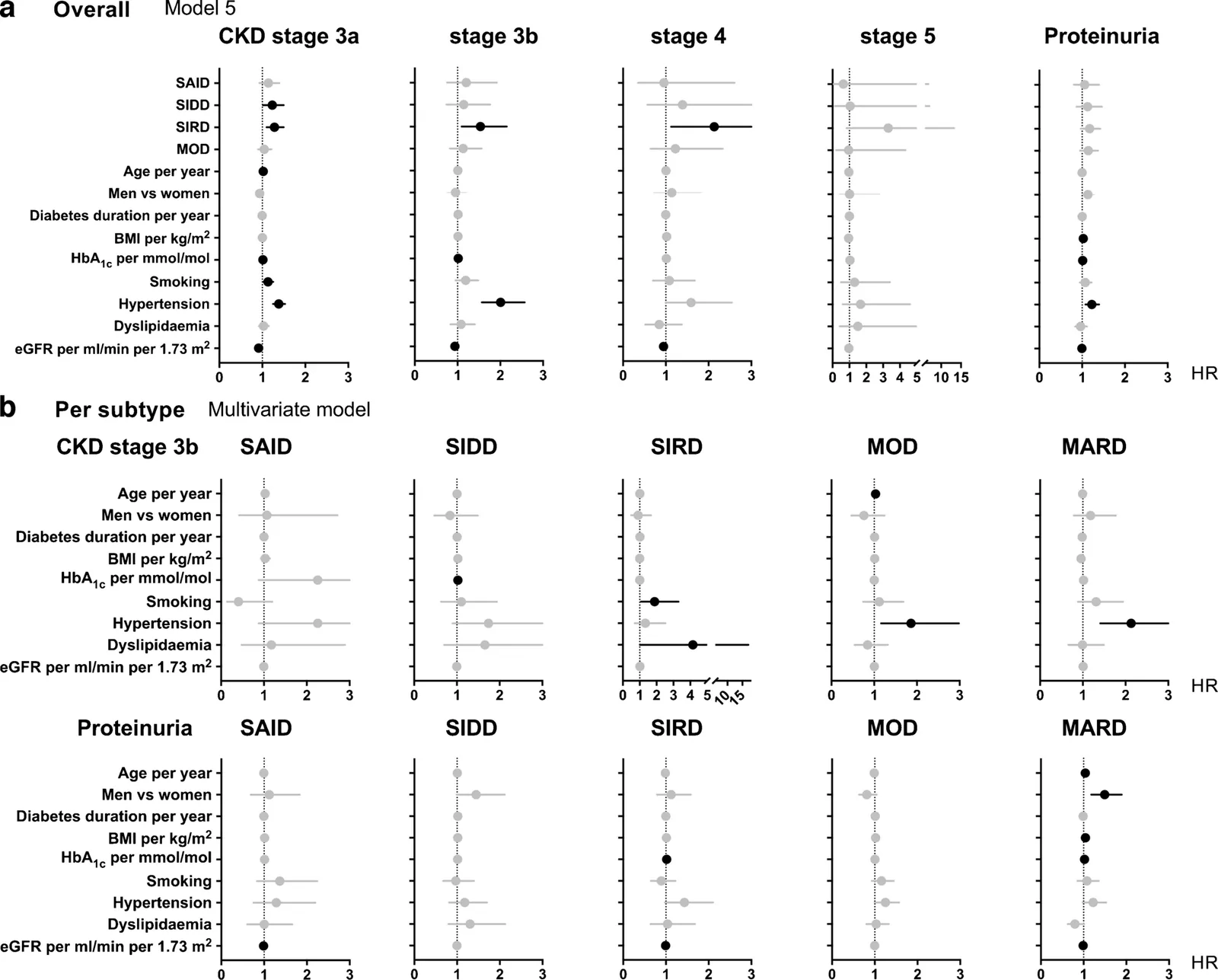
Multivariate Cox proportional hazards model for the development of renal events (**a**) overall and (**b**) per subtype. (**a**) In the overall analysis, HRs in Model 5 of Table 1 for stage 3a (eGFR<60 ml/min per 1.73 m^2^), stage 3b (eGFR<45 ml/min per 1.73 m^2^), stage 4 (eGFR<30 ml/min per 1.73 m^2^), stage 5 (eGFR<15 ml/min per 1.73 m^2^) and proteinuria are shown. (**b**) Per subtype, HRs in the multivariate model for stage 3b (eGFR<45 ml/min per 1.73 m^2^) and proteinuria are shown among subtypes SAID, SIDD, SIRD, MOD and MARD. Analyses for stages 3a, 3b, 4 and 5 were performed using Dataset B (*n*=7146) and those for proteinuria using Dataset C (*n*=5844) in a Japanese diabetes cohort database (J-DREAMS). Circles and bars indicate HRs and 95% CIs. Black circles indicate statistically significant HRs, while the grey circles indicate non-significant HRs

The Cox proportional hazards models were produced by correcting for representative DKD risk factors without (Models 1, 2, 3 and 4; Table 1) or with (Model 5; Table 1, Fig. 2a) Ahlqvist’s variables (BMI and HbA_1c_). In the fully adjusted model (Model 5), SIRD demonstrated significant associations with CKD stage 3a (HR 1.28; 95% CI 1.10, 1.50; *q*=0.012), stage 3b (HR 1.53; 95% CI 1.10, 2.15; *q*=0.06) and stage 4 (HR 2.13; 95% CI 1.12, 4.05; *q*=0.085) for multiple testing using the Benjamini–Hochberg procedure (*q*<0.1). SIDD showed a borderline significant association with stage 3a (HR 1.23; 95% CI 1.01, 1.50; *q*=0.138). For stage 5, the SIRD subtype showed elevated but non-significant HRs (HR 3.31; 95% CI 0.82, 13.3; *q*=0.269). For stage 3b, we found the following changes in HRs from Model 2 (excluding both HbA_1c_ and BMI) to Model 5 (fully adjusted model including Ahlqvist’s variables, BMI and HbA_1c_): SAID from 1.32 to 1.20 (9.1% reduction), SIDD from 1.68 to 1.14 (32.1% reduction), SIRD from 1.60 to 1.53 (4.4% reduction) and MOD from 1.16 to 1.13 (2.6% reduction). However, statistical significance was maintained for all subtypes, suggesting that overfitting in Model 5 due to the duplicate use of Ahlqvist’s variables was minimal. In assessments of multicollinearity VIF analysis, all VIF values were less than 5, showing no severe multicollinearity (Table 1).

In the assessment of Cox proportional hazards model assumptions, global Schoenfeld residuals tests indicated statistically significant violations for stage 3a (*p*<0.001), stage 3b (*p*=0.022) and proteinuria (*p*=0.023). However, for the diabetes subtype variables (SAID, SIDD, SIRD, MOD), which were the primary focus of our analysis, individual tests demonstrated satisfaction of the proportional hazards assumption across all outcomes (stage 3a: *p*=0.131–0.440; stage 3b: *p*=0.104–0.958; proteinuria: *p*=0.075–0.980). The global test violations were primarily attributable to continuous variables (age, eGFR, HbA_1c_) and might not materially affect the interpretation of HR comparisons between subtypes (ESM Table 3).

For the sensitivity analysis, an alternative clustering was performed using Ahlqvist's variables plus classical DKD risk factors, which resulted in five distinct clusters (ESM Fig. 6). The baseline characteristics of these alternative subtypes are shown in ESM Table 4. Based on the Cox proportional hazards model using the combined variables (ESM Table 5, ESM Fig. 7), the discriminative abilities were as follows: stage 3a, Ahlqvist’s classification *p*<0.001 vs DKD-specific classification *p*<0.001; stage 3b, Ahlqvist’s classification *p*<0.001 vs DKD-specific classification *p*=0.040; stage 4, Ahlqvist’s classification *p*<0.001 vs DKD-specific classification *p*=0.620; and stage 5, Ahlqvist’s classification *p*=0.091 vs DKD-specific classification *p*=0.290. The predictive accuracy using the C-index yielded for G3a events: SIRD vs others: C-index 0.640; DKD vs others: C-index 0.637, difference: −0.0025, *z* score −0.678, *p* value 0.498, which was not statistically significant.

### Sub-analysis in the Cox proportional hazards model

As shown in ESM Table 6, SIRD had a higher HR in ≥65 years, men, 2–10 years of diabetes duration and a BMI of <18.5 and ≥25 for stage 3b events.

### Cox proportional hazards model per subtype

The baseline characteristics of individuals with and without eGFR and proteinuria events among diabetes subtypes are shown in ESM Table 7. The Cox proportional hazards model for stage 3b and proteinuria per subtype is shown in Table 2 and Fig. 2b. To statistically validate the subtype-specific effects suggested by stratified analyses, we performed formal testing of interaction terms (ESM Table 8). Significant interaction terms were observed for stage 3b events: MOD × age (*p*=0.029), MOD × sex (*p*=0.037), MOD × HbA_1c_ (*p*=0.017) and SAID × smoking (*p*=0.018). For proteinuria, significant interaction terms included SAID × age (*p*=0.040), SAID × HbA_1c_ (*p*=0.011), SAID × dyslipidaemia (*p*=0.010), SIDD × sex (*p*=0.025) and SIRD × diabetes duration (*p*=0.030). BMI interaction terms were not statistically significant across all subtypes (all *p*>0.30).

**Table 2:** Per subtype Cox proportional hazards model for eGFR and proteinuria events

Variable	SAID		SIDD		SIRD		MOD		MARD	
	HR (95% CI)	*p* value	HR (95% CI)	*p* value	HR (95% CI)	*p* value	HR (95% CI)	*p* value	HR (95% CI)	*p* value
CKD G3b multivariate model										
Age per year	1.02 (0.99, 1.07)	0.179	1.00 (0.98, 1.03)	0.797	1.00 (0.98, 1.03)	0.769	1.03 (1.01, 1.06)	0.019	0.99 (80.97, 1.02)	0.615
Men vs women	1.07 (0.42, 2.72)	0.889	0.84 (0.47, 1.49)	0.546	0.90 (0.49, 1.67)	0.749	0.76 (0.46, 1.24)	0.271	1.18 (0.79, 1.77)	0.419
Diabetes duration per year	1.00 (0.96, 1.04)	0.994	1.00 (0.98, 1.03)	0.784	1.01 (0.98, 1.04)	0.477	1.01 (0.98, 1.03)	0.501	0.98 (0.96, 1.01)	0.246
BMI per kg/m^2^	1.03 (0.93, 1.14)	0.600	1.02 (0.97, 1.08)	0.397	1.00 (0.95, 1.04)	0.871	1.01 (0.97, 1.06)	0.578	0.96 (0.91, 1.01)	0.131
HbA_1c_ per mmol/mol	1.01 (0.98, 1.04)	0.626	1.02 (1.00, 1.04)	0.036	1.00 (0.98, 1.03)	0.821	1.00 (0.98, 1.03)	0.990	1.02 (0.99, 1.04)	0.162
Current or ex-smoking vs never smoking	0.40 (0.13, 1.19)	0.101	1.10 (0.63, 1.94)	0.732	1.88 (1.07, 3.29)	0.027	1.12 (0.74, 1.69)	0.590	1.31 (0.88, 1.94)	0.177
Hypertension yes vs no	2.26 (0.88, 5.80)	0.092	1.74 (0.90, 3.36)	0.100	1.33 (0.70, 2.53)	0.381	1.86 (1.16, 2.98)	0.010	2.13 (1.41, 3.22)	<0.001
Dyslipidaemia yes vs no	1.17 (0.48, 2.89)	0.728	1.66 (0.70, 1.94)	0.249	4.15 (1.01, 17.0)	0.048	0.84 (0.54, 1.32)	0.458	0.99 (0.66, 1.49)	0.968
eGFR per ml/min per 1.73 m^2^	1.00 (0.97, 1.02)	0.728	1.00 (0.98, 1.01)	0.451	1.01 (1.00, 1.02)	0.120	1.00 (0.99, 1.01)	0.577	1.01 (1.00, 1.01)	0.202
Proteinuria multivariate model										
Age per year	0.99 (0.97, 1.01)	0.471	1.00 (0.99, 1.02)	0.648	0.99 (0.97, 1.00)	0.055	0.98 (0.97, 1.00)	0.053	1.04 (1.02, 1.06)	<0.001
Men vs women	1.12 (0.68, 1.83)	0.652	1.45 (0.99, 2.12)	0.057	1.12 (0.79, 1.59)	0.523	0.81 (0.63, 1.04)	0.104	1.49 (1.18, 1.89)	0.001
Diabetes duration per year	0.99 (0.97, 1.02)	0.684	1.01 (1.00, 1.03)	0.140	1.00 (0.98, 1.02)	0.819	1.01 (1.00, 1.02)	0.169	0.99 (0.97, 1.00)	0.151
BMI per kg/m^2^	1.01 (0.96, 1.07)	0.668	1.02 (0.98, 1.05)	0.348	1.01 (0.99, 1.04)	0.382	1.02 (1.00, 1.04)	0.070	1.04 (1.01, 1.08)	0.013
HbA_1c_ per mmol/mol	1.01 (0.99, 1.03)	0.205	1.01 (1.00, 1.03)	0.113	1.01 (1.00, 1.03)	0.043	1.01 (0.99, 1.02)	0.423	1.02 (1.01, 1.03)	0.001
Current or ex-smoking vs never smoking	1.37 (0.83, 2.24)	0.218	0.97 (0.67, 1.39)	0.863	0.89 (0.65, 1.22)	0.474	1.16 (0.93, 1.44)	0.195	1.08 (0.86, 1.35)	0.526
Hypertension yes vs no	1.28 (0.75, 2.20)	0.362	1.18 (0.81, 1.70)	0.388	1.43 (0.97, 2.10)	0.067	1.25 (0.99, 1.57)	0.055	1.22 (0.98, 1.53)	0.078
Dyslipidaemia yes vs no	1.00 (0.61, 1.66)	0.993	1.30 (0.80, 2.13)	0.293	1.04 (0.64, 1.68)	0.879	1.03 (0.80, 1.33)	0.805	0.80 (0.63, 1.01)	0.060
eGFR per ml/min per 1.73 m^2^	0.99 (0.97, 1.00)	0.037	1.00 (0.99, 1.00)	0.380	0.99 (0.98, 1.00)	0.030	1.00 (0.99, 1.00)	0.379	0.99 (0.98, 1.00)	0.001

For stage 3b, no factors in SAID had a significant multivariate HR, while HbA_1c_ in SIDD, smoking in SIRD, age and hypertension in MOD and hypertension in MARD had significant multivariate HRs. For proteinuria, none of the factors had a significant HR in SAID, whereas male sex in SIDD, hypertension in SIRD and MOD and age, male sex, BMI and HbA_1c_ in MARD had significant multivariate HRs.

### Cluster reproducibility analysis

Among 3952 participants in Dataset E with clustering variables available at both baseline and follow-up (mean interval: 5.0±1.2 years), the distribution of baseline clusters was: SIDD 20.5% (*n*=808), SIRD 16.5% (*n*=653), MOD 29.4% (*n*=1163) and MARD 33.6% (*n*=1328). Clinical characteristics of Dataset E participants were comparable to those of Dataset A (ESM Table 9).

Cluster reproducibility varied largely across subtypes (ESM Fig. 8). MARD demonstrated the highest reproducibility with 94.8% of participants maintaining their cluster assignment, followed by SIDD (74.1%), MOD (69.7%) and SIRD (31.4%). The SIRD subtype showed the lowest reproducibility, with 68.6% of participants migrating to other clusters over the follow-up period. Among baseline SIRD participants (*n*=653), the most common migration was to MOD (38.4%, *n*=251), followed by MARD (26.3%, *n*=172), with only 31.4% (*n*=205) maintaining SIRD classification and 3.8% (*n*=25) migrating to SIDD. Similar patterns were observed for other baseline clusters, with MOD showing the second-highest migrating rate to MARD (24.0%) while maintaining 69.7% reproducibility. Among baseline SIRD participants who transitioned to other subtypes or remained in the same subtype, stage 3b incidence rates tended to vary by final cluster assignment (*p*=0.330): 18.2% (2/11) of those who transitioned to SIDD, 16.8% (18/107) of those who maintained SIRD, 10.3% (16/155) of those who transitioned to MOD and 9.8% (8/82) of those who transitioned to MARD.

## Discussion

In this study, we used a Japanese diabetes cohort study (J-DREAMS) [14] to examine whether data-driven clustering is useful for stratifying the risk of DKD or ESKD. This study had three main findings. First, diabetes clustering using the *k*-means method was able to classify individuals in the cohort into five subtypes, and the clinical characteristics of the five subtypes were similar to those previously reported [8–11]. Second, Kaplan–Meier curve analysis showed that events for stages 3a, 3b and 4 and proteinuria were the highest in the SIRD subtype, and the Cox proportional hazards model showed that the SIRD subtype had significant HRs for stages 3a, 3b and 4 after correction for multiple testing. Third, each subtype had diverse combinations of factors associated with stage 3b and proteinuria events in the Cox proportional hazards models. We found that the data-driven subtypes of Ahlqvist et al [8] were reproducible in a nationwide Japanese diabetes database of routine clinical practice and that the risk of developing DKD or ESKD differed greatly among the five subtypes. This study is the first to show that the combinations of risk factors for DKD and ESKD differ for each diabetes subtype, suggesting the possibility of personalised prevention and treatment strategies for each subtype.

Cluster analysis using the *k*-means method divides individuals of several races into similar subtypes: SAID, SIDD, SIRD, MOD and MARD [8–11, 22, 23]. In Japanese individuals, analyses have been conducted only on relatively small numbers [11, 12, 24, 25]. However, this study confirmed that individuals with diabetes in a nationwide database, J-DREAMS, can also be divided into five subtypes, suggesting that the five subtypes are universally applicable to the Japanese population with diabetes.

According to the Kaplan–Meier curve analysis, stages 3b and 4 events were most frequent in the SIRD subtype from the J-DREAMS database, mimicking previous reports [8, 10]. In clinical practice, physicians commonly intervene in blood glucose, blood pressure, lipid, obesity and smoking to prevent the onset of DKD/ESKD. Therefore, the presence or absence of these risk factors at baseline is critical for predicting the incidence of DKD/ESKD. However, the analysis of HR for SIRD in previous reports did not adjust for these confounding factors, limiting the application of this classification in clinical practice.

In a report using multivariate analysis, SIRD showed a significant HR in Cox regression analysis but adjusted only for sex, age at onset and baseline eGFR: stage 3a HR 1.56 (95% CI 1.34, 1.82), stage 3b HR 1.86 (1.44, 2.41), stage 5 HR 2.00 (1.16, 3.49) and macroalbuminuria HR 2.45 (1.62, 4.71) [8]. In another study, Cox regression analysis adjusted for baseline eGFR showed no significant HR (stage 3a 1.11 [0.53–2.35]) [10]. Previously, to our knowledge, we performed the only multivariable analysis adjusting for clinically important confounders of the incidence of DKD (age at onset, sex, history of diabetes, BMI, HbA_1c_, smoking, hypertension, dyslipidaemia and retinopathy) [2, 3, 7] and reported that SIRD still had a significant HR of 2.19 (1.44–3.44) [11]. However, the results of the above multivariate-adjusted analysis have not yet been verified.

The current study validated previous findings that SIRD is a risk factor for stage 3b and 4, even after correcting for multiple confounders of the incidence of DKD. In addition, the results obtained from a large nationwide database suggest greater generalisability than those of a previous report based on a small single-hospital population [11]. In terms of cluster separation, DKD-specific classification showed less clear separation compared with Ahlqvist classification. These results indicate that DKD-specific clustering did not improve predictive accuracy compared with Ahlqvist classification and demonstrated inferior discriminative ability in advanced CKD. Despite having fewer variables, the Ahlqvist classification maintains consistent discriminative ability in DKD prediction, suggesting its high clinical utility.

In the sub-analysis, SIRD was associated with a higher HR for stage 3b events (ESM Table 6) in individuals aged ≥65 years, male individuals, those with diabetes duration of 2–10 years, those with a BMI of ≤18.5 and ≥25 and those with HbA_1c_<63 mmol/mol (8.0%), and with a higher HR for proteinuria events in individuals aged <65 years, female individuals and those with diabetes duration of 2–10 years. The results of the sub-analysis suggest that the effect of SIRD on DKD varies according to clinical features such as age, sex, diabetes duration and HbA_1c_.

This study revealed diverse combinations of risk factors for stage 3b and proteinuria events per subtype, with formal testing of interaction terms providing statistical validation for some of the observed differences. The significant interaction terms observed, particularly for the MOD subtype with age, sex and HbA_1c_, and for the SAID subtype with multiple risk factors, provide statistical evidence for genuine subtype-specific effects that may guide personalised intervention strategies. However, the finding that many risk factors, including BMI and hypertension, showed consistent effects across subtypes (non-significant interaction terms) indicates that core DKD prevention strategies remain universally important. To our knowledge, this study is the first both to examine the risk of developing DKD for each diabetes subtype and to formally test interaction terms to validate subtype-specific risk factor effects.

The reason the risk factors for stage 3b and proteinuria events partially differ for each subtype is unclear from the current results. In other words, it is unclear why common risk factors for the development and progression of DKD (age, male sex, obesity, smoking, elevated glucose levels and hypertension) [7] affect each subtype unequally. Diabetes subtype is an innate classification determined when a person is diagnosed with diabetes, and genetic factors may be at play [8, 26–28]. Although previous reports indicated no strong consistency over time, we showed that a large proportion of individuals, except for the undecidable group (14.2%), showed higher consistency over time [12]. Ahlqvist and colleagues [8, 26], Udler and colleagues [27, 28] and our unpublished observations (M. Shimabukuro et al, unpublished data) have shown that the genetic risk score for diabetes-related traits differs among diabetes subtypes. The five diabetes subtypes, which have different genetic backgrounds, exhibit different clinical characteristics. Furthermore, the response to glucose-lowering medications differs among the subtypes. For example, the glucose-lowering efficacy of insulin, metformin, dipeptidyl peptidase 4 inhibitors (DPP4i) and sodium–glucose cotransporter 2 inhibitors (SGLT2i) [8, 10, 29], as well as the non-protective effects of canagliflozin [29], differs between subtypes. The combined genetic background, clinical characteristics and drug responsiveness of each subtype may result in different risk factors for G3b or proteinuria. These results suggest that the effects of interventions on these risk factors differ between subtypes. This notion warrants investigation into whether intervention strategies should be modified for each subtype [13].

This study has several limitations. First, this retrospective analysis of the J-DREAMS database may be associated with an immeasurable selection bias [14]. The use of EMR data, including measurement errors (e.g. accuracy of diagnostic and procedural codes and prescription drug codes), has limited generalisability. Because all participants in J-DREAMS were treated at referral centres (e.g. hospital outpatient clinics), this study may have included individuals with more advanced type 1 or type 2 diabetes rather than the general population of individuals with diabetes treated by general practitioners or family doctors. Second, the study population consisted solely of Japanese individuals, which may limit the generalisability of our findings to other ethnic groups or populations. Third, proteinuria was determined using semi-quantitative dipstick urinalysis, which could introduce random measurement errors and regression dilution biases, potentially leading to an underestimation of the association. However, previous reports indicate that most (80–98%) cases with proteinuria graded as 1+ or higher on dipstick testing are also positive for albuminuria [30]. Thus, we defined DKD based on the assumption that proteinuria graded 1+ on a dipstick urine test corresponded to an albumin/creatinine ratio 3.4–34 mg/mmol (30–300 mg/g). Fourth, fasting sampling of plasma glucose and C-peptide had not been completely guaranteed, limiting our calculation on HOMA2-IR and HOMA2-β. In addition, the validity of the HOMA indices should be carefully considered in individuals with a lower BMI, lower beta cell function and high fasting glucose levels, such as those with SAID and SIDD subtypes with insulin secretory defects [31]. We manually omitted FPG levels <3.9 mmol/l or >11.1 mmol/l to minimise adverse effects on the calculations of the HOMA indices. Fifth, the substantial reduction in the number at risk during the later follow-up period (particularly after 7 years) can lead to statistical instability in Kaplan–Meier curve estimates for CKD outcomes. The number at risk decreased by approximately 80% between 7 and 8 years across most groups (e.g. MARD stage 4: 173 to 33 participants), creating small sample effects where individual events can disproportionately influence cumulative incidence estimates. This statistical artefact may result in misleading cumulative incidence patterns that do not reflect the true underlying risk, as demonstrated by the substantially lower actual event rates observed in the Cox proportional hazards models (e.g. 1.3% for MARD stage 4 events), which provide more stable time-adjusted risk estimates. An experimental Kaplan–Meier model censored at 7 years showed better discrimination between subtype curves (ESM Fig. 9). Finally, our cluster reproducibility analysis revealed that substantial numbers of individuals, particularly for SIRD, were assigned to other subtypes over approximately 5 years and that the incidence of DKD may differ among those with SIRD maintained or transitioned. As discussed elsewhere [9, 12], it is not possible to draw any conclusions at this point regarding the consistency of subtypes over time and DKD prognosis. This issue should be considered in future studies, which may clarify the phenotype and pathophysiology of the undecidable subtype [12].

In conclusion, the results showed the possibility of forecasting the probability of developing DKD or ESKD using data-driven diabetes subtyping in a large Japanese diabetic population and the diverse combinations of factors predisposing each diabetes subtype to the development of DKD/ESKD. These results suggest that using data-driven diabetes subtyping in daily clinical practice to assess the likelihood of developing DKD/ESKD and mitigating predisposing factors in each subtype is useful for developing personalised prevention strategies.

## Supplementary Information

Below is the link to the electronic supplementary material.

ESM (PDF 2.53 MB)

## Data Availability

The datasets generated during and/or analysed in the current study are not publicly available but are available from the corresponding author upon reasonable request.

## Abbreviations

CKD: Chronic kidney disease
DKD: Diabetic kidney disease
EMR: Electronic medical record
ESKD: End-stage kidney disease
FPG: Fasting plasma glucose
J-DREAMS: The Japan Diabetes compREhensive database project based on Advanced electronic Medical record System
MARD: Mild age-related diabetes
MOD: Mild obesity-related diabetes
SAID: Severe autoimmune diabetes
SIDD: Severe insulin-deficient diabetes
SIRD: Severe insulin-resistant diabetes
VIF: Variance inflation factor
