# Supplementary material for: Diverse combination of factors associated with the development of diabetic kidney disease among data-driven diabetes subtypes: analysis of the J-DREAMS registry
Source: Diabetologia. 2025 Nov 17;69(4):883–99. doi: 10.1007/s00125-025-06594-1 (PMC12957631; doi:10.1007/s00125-025-06594-1)
Supplement: Supplementary file 1 — ESM (PDF 4.53 MB) [file 125_2025_6594_MOESM1_ESM.pdf]

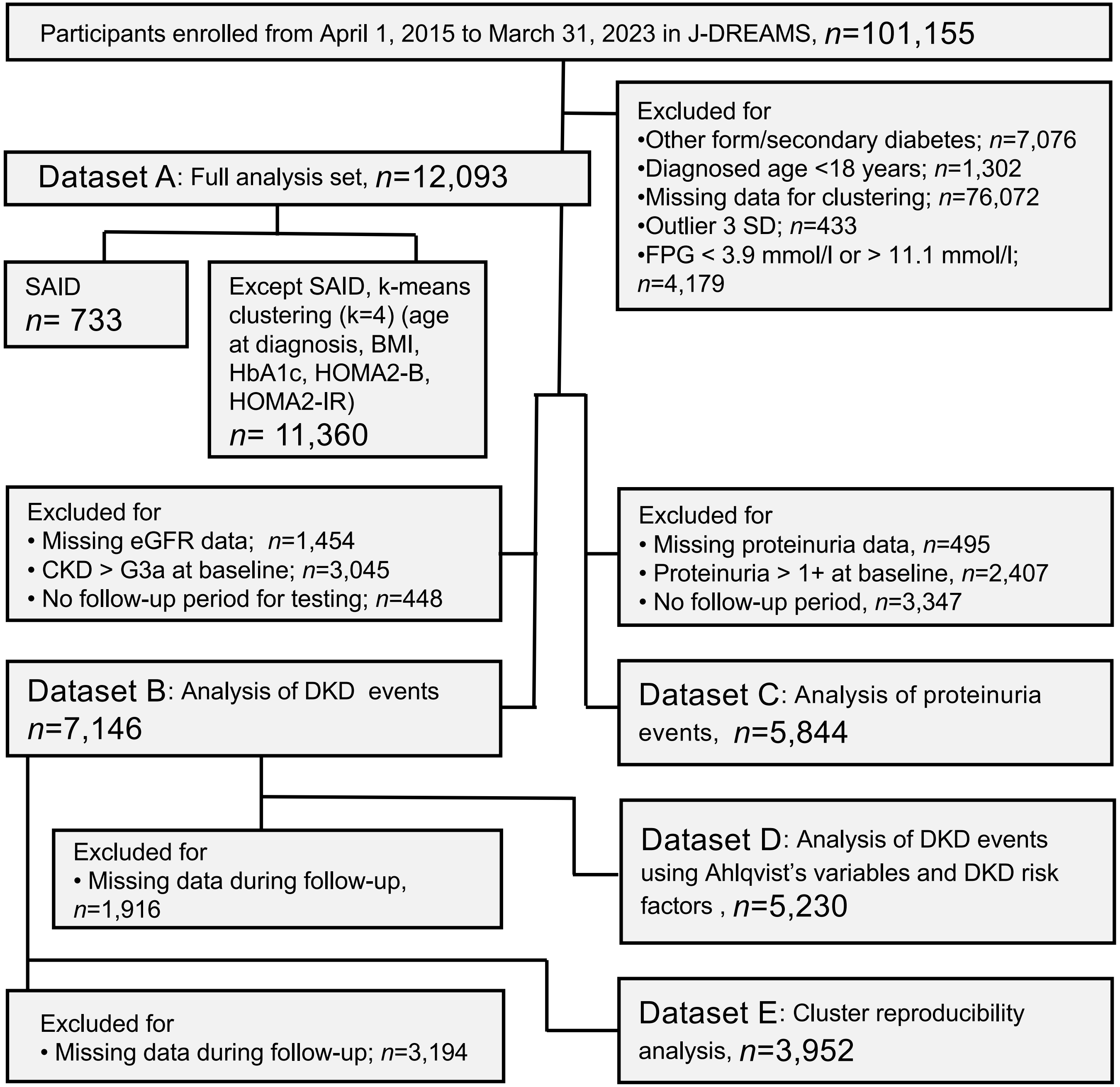

ESM Fig. 1 Flow chart for participants

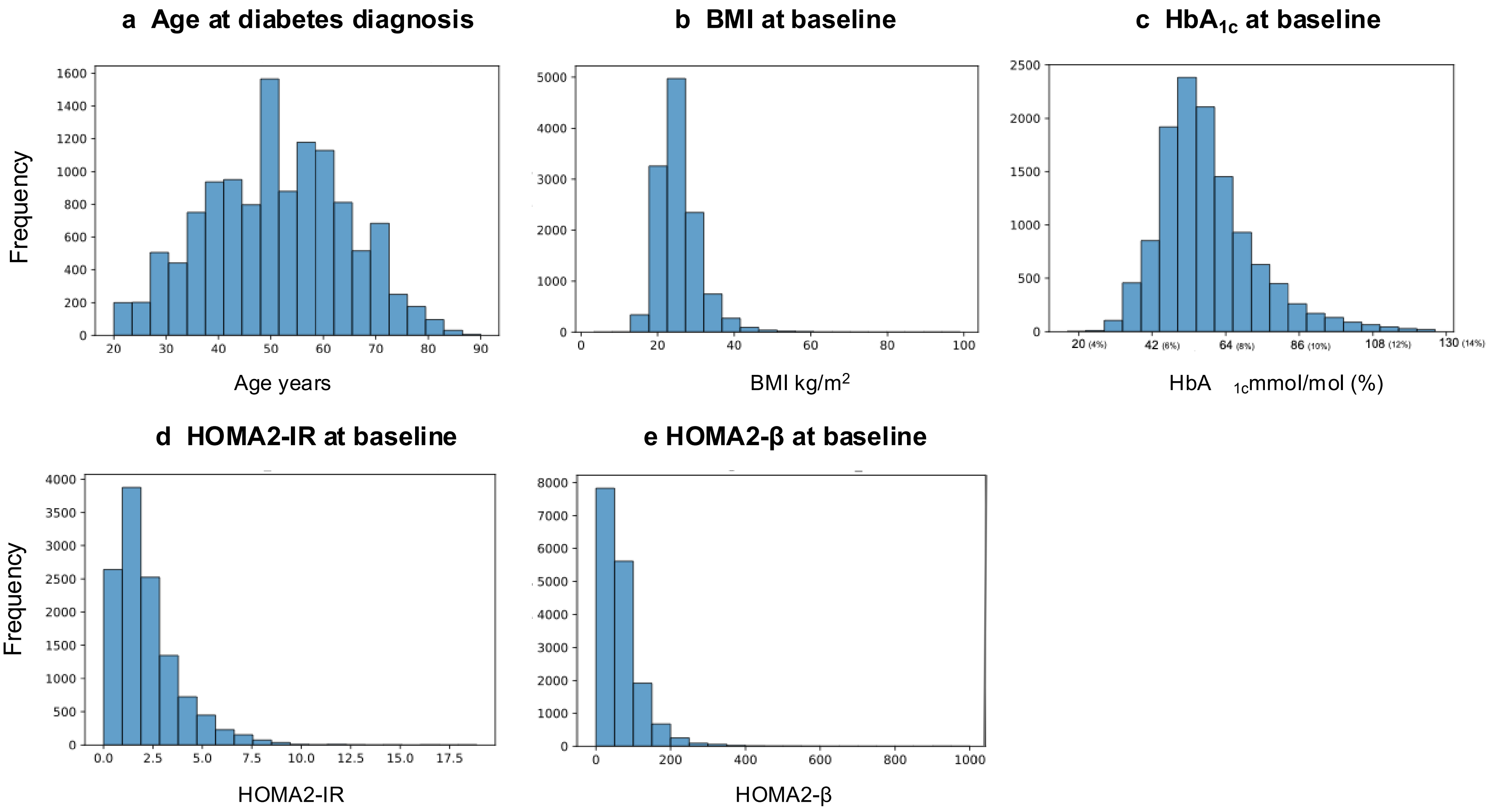

ESM Fig. 3 Distribution of the five Ahlqvist's variables, except GAD antibody levels, in the J-DREAMS resistry (Dataset A, *n*=12,093)

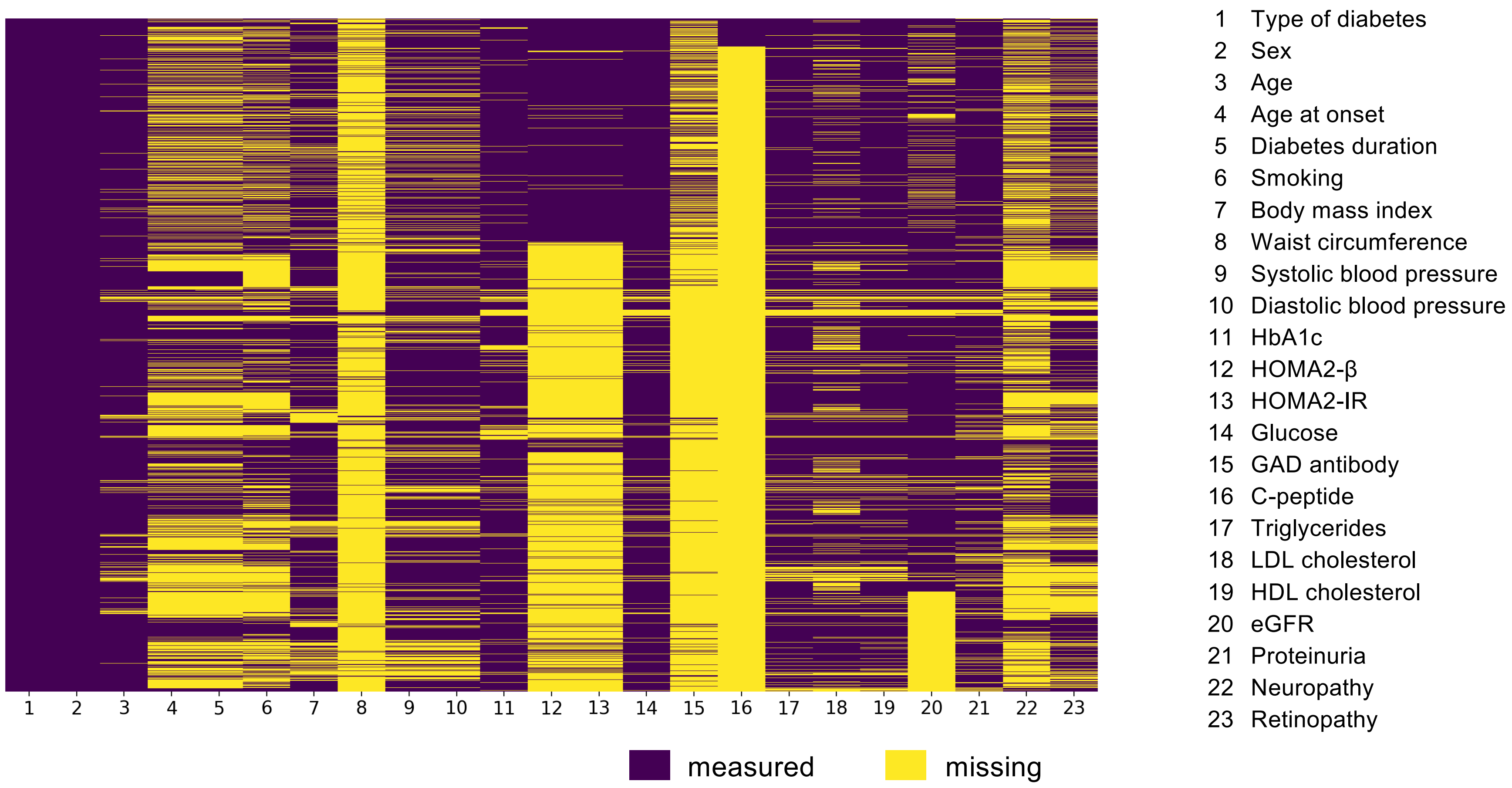

ESM Fig. 2 Heatmap for data missing pattern

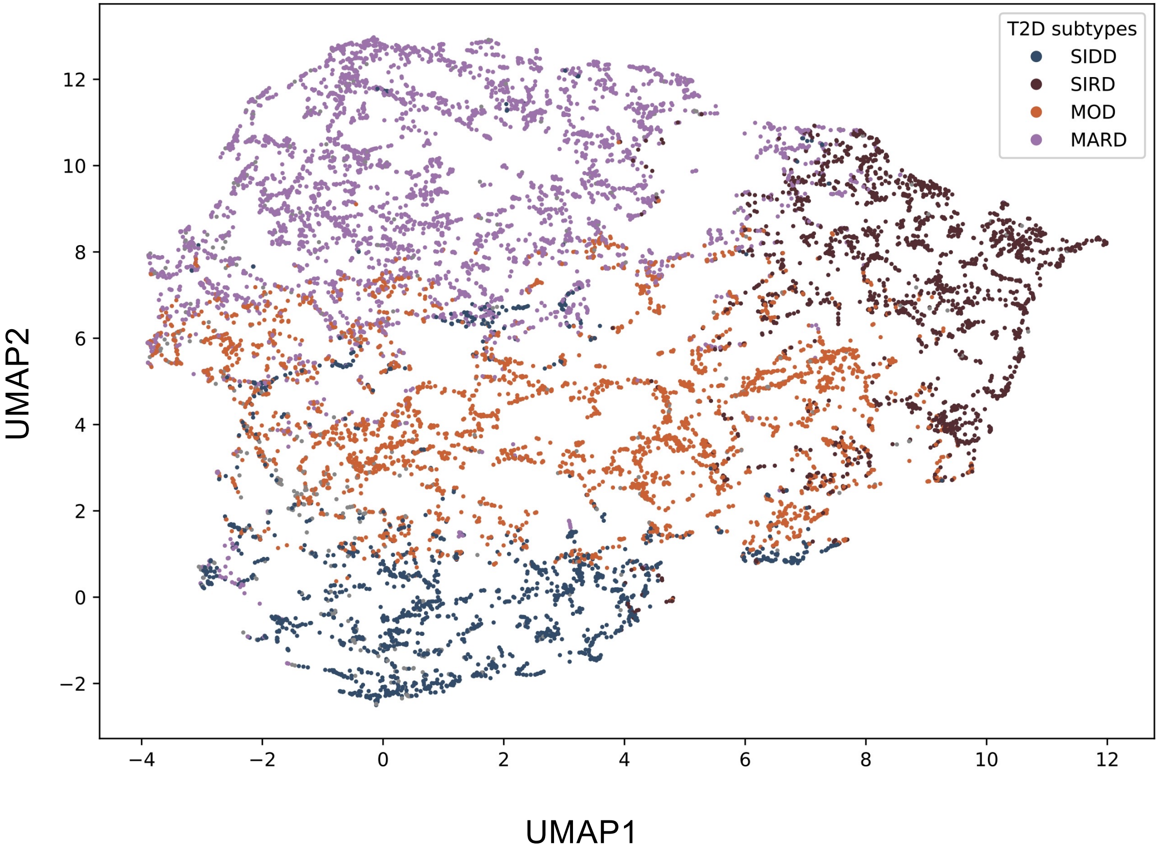

ESM Fig. 4 Two-dimensional visualisation of the proximity matrix of individuals from Dataset A, excluding SAID (*n*=11,360). Colours indicate SIDD (severe insulin-deficient diabetes, blue dots), SIRD (severe insulin-resistant diabetes, red dots), MOD (mild obesity-related diabetes, orange dots), and MARD (mild age-related diabetes, purple dots) in subtypes of type 2 diabetes (T2D). UMAP: uniform manifold approximation and projection.

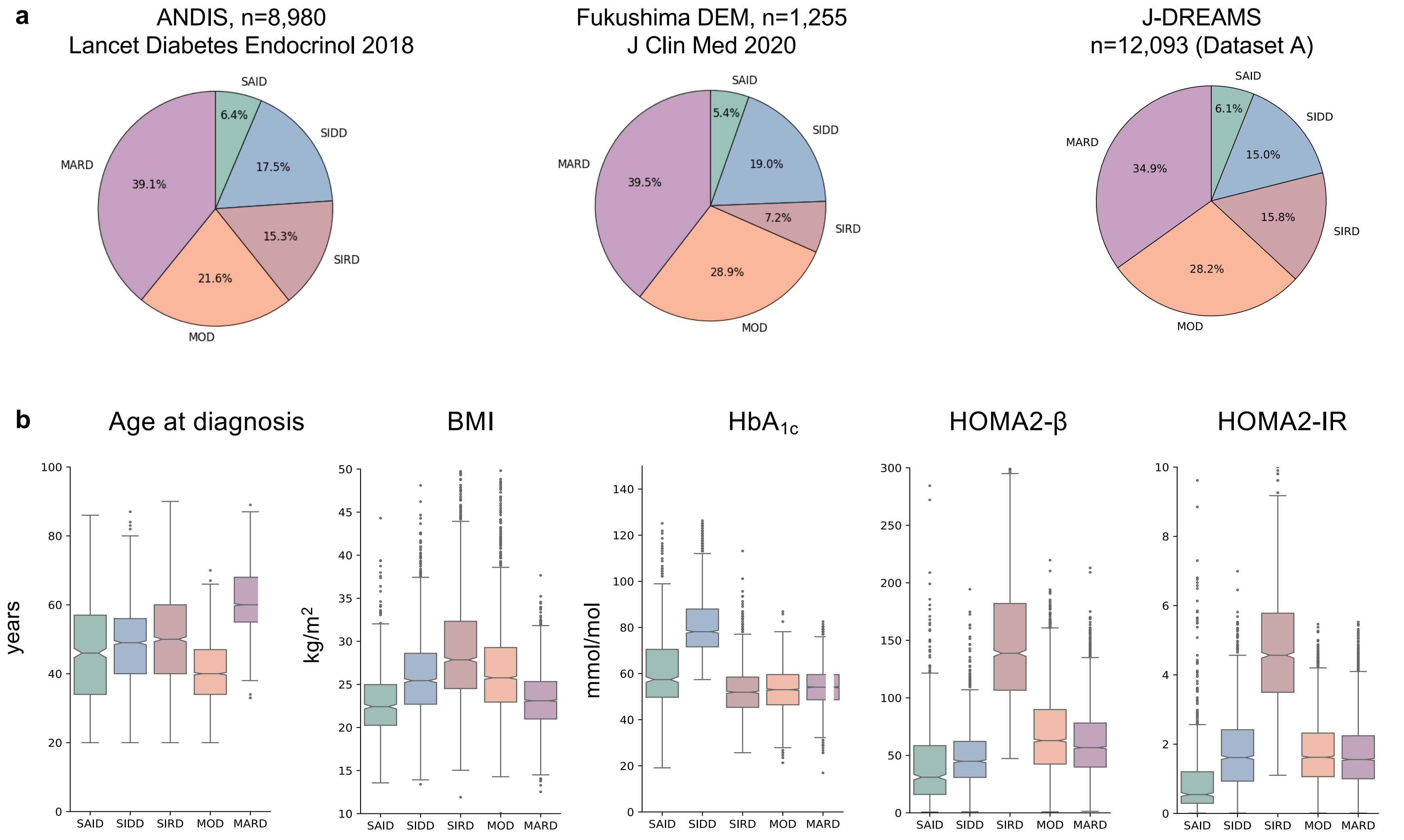

ESM Fig. 5 (**a**) Distributions (%) of diabetes subtypes in ANDIS [8], Fukushima DEM [11], and J-DREAMS. (**b**) Distributions of age at diagnosis, BMI, HbA_{1c}, HOMA2-β, and HOMA2-IR at baseline in J-DREAMS (Dataset A). SAID: severe autoimmune diabetes; SIDD: severe insulin-deficient diabetes; SIRD: severe insulin-resistant diabetes; MOD: mild obesity-related diabetes; MARD: mild age-related diabetes; BMI: body mass index; HbA_{1c}: hemoglobin A_{1c}; HOMA2-β: homoeostatic model assessment 2 estimates of β-cell function; HOMA2-IR: homoeostatic model assessment 2 estimates of insulin resistance. Whiskers show median with interquartile range (IQR) and bars indicate 95% confidence interval (CI).

ESM Table 1. Baseline characteristics of Ahlqvist's diabetes subtypes in Dataset A

Dataset A

Variables	All *n* =12,093	SAID *n* =733 (6.1%)	SIDD *n* =1,814 (15.0%)	SIRD *n* =1,914 (15.8%)	MOD *n* =3,413 (28.2%)	MARD *n* =4,219 (34.9%)	*p* value
Women, %	42	54	47.400	34.600	36.300	44.200	<0.001
Age, years	62 ± 14	60 ± 16	62 ± 13	59 ± 15	55 ± 14	71 ± 8	<0.001
(min - max)	(20 - 97)	(21 - 94)	(21 - 95)	(20 - 93)	(20 - 92)	(39 - 97)	
Age at diagnosis, years	51 ± 13	46 ± 15	48 ± 12	50 ± 13	40 ± 8	61 ± 8	<0.001
Diabetes duration,years	12 ± 10	10 ± 10	13 ± 11	9 ± 9	15 ± 12	10 ± 8	<0.001
Smoking, %	51.700	45.400	51.300	46.600	54.000	48.500	<0.001
Anthropometry							
BMI, kg/m^2	25.5 ± 5.4	23.0 ± 4.3	26.0 ± 4.8	29.2 ± 6.8	26.6 ± 5.5	23.2 ± 3.3	<0.001
Waist circumference, cm	92 ± 26	83 ± 10	94 ± 15	102 ± 18	96 ± 43	87 ± 11	<0.001
Systolic blood pressure, mmHg	129 ± 19	124 ±16	130 ± 19	131 ± 18	128 ± 23	130 ± 17	<0.001
Diastolic blood pressure, mmHg	75 ± 19	74 ± 30	76 ± 26	77 ± 13	76 ± 21	73 ± 12	<0.001
Biochemistry							
HbA_{1c}							
%	7.5 ± 1.3	7.8 ± 1.5	9.6 ± 1.2	7.0 ± 1.0	6.9 ± 0.9	7.1 ± 0.8	<0.001
mmol/mol	58.0 ± 14.5	61.4 ± 16.5	81.3 ± 13.1	52.8 ± 11.0	52.4 ± 9.5	54.1 ± 8.4	<0.001
HOMA2-β	74.7 ± 51.6	43.7 ± 43.8	48.7 ± 25.0	151.8 ± 64.1	68.5 ± 35.0	61.2 ± 28.7	<0.001
HOMA2-IR	2.19 ± 1.70	1.01 ± 1.30	1.77 ± 1.10	4.88 ± 2.03	1.74 ± 0.93	1.71 ± 0.97	<0.001
Fasting plasma glucose							
mg/dL	136 ± 30	133 ± 34	152 ± 30	131 ± 31	132 ± 29	136 ± 27	<0.001
mmol/l	7.6 ± 1.7	7.4 ± 1.9	8.4 ± 1.7	7.3 ± 1.7	7.1 ± 1.6	7.5 ± 1.5	<0.001
Triacylglycerol, mmol/l	1.73 ± 1.38	1.25 ± 0.99	1.95 ± 1.89	2.16 ± 1.43	1.70 ± 1.29	1.51 ± 1.14	<0.001
HDL cholesterol, mmol/l	1.42 ± 0.41	1.73 ± 0.52	1.34 ± 0.39	1.27 ± 0.36	1.40 ± 0.39	1.45 ± 0.41	<0.001
LDL cholesterol, mmol/l	2.69 ± 0.88	2.72 ± 0.83	2.87 ± 0.98	2.77 ± 0.93	2.69 ± 0.88	2.61 ± 0.78	<0.001
eGFR, ml/min per 1.73m^2	73 ± 25	79 ± 25	77 ± 27	69 ± 29	78 ± 28	68 ± 20	<0.001
Complications							
CKD stage 3a, %	25.2	14.2	20.8	32.8	20.5	29.3	<0.001
Proteinuria, %	19.9	11.6	25.4	25.7	19.8	16.4	<0.001
Hypertension, %	64.2	40.1	65.9	72.0	60.0	67.7	<0.001
Dyslipidemia, %	77.9	55.8	82.6	87.5	77.2	76.1	<0.001
Polyneuropathy, %	31.3	26.3	41.3	29.8	29.6	29.7	<0.001
Retinopathy, %	20.9	19.9	30.1	14.8	24.7	17.0	<0.001
Medications							
Metformin, %	38.4	15.8	49.7	36.9	44.3	33.4	<0.001
Insulin, %	35.4	79.0	57.4	19.4	33.1	27.3	<0.001
Observation period, years	2.4 ± 2.4	2.9 ± 2.3	2.0 ± 2.1	2.4 ± 2.1	2.7 ± 2.2	2.7 ± 2.2	<0.001
(min - max)	(0 - 8.4)	(0 - 8.4)	(0 - 8.4)	(0 - 8.4)	(0 - 8.4)	(0 - 8.4)	

Values are presented as mean ± SD, or n (%). *P* values were obtained by one-way ANOVA or Chi-square test. SAID: severe autoimmune diabetes; SIDD: severe insulin-deficient diabetes; SIRD: severe insulin-resistant diabetes; MOD: mild obesity-related diabetes; MARD: mild age-related diabetes; BMI: body mass index; HbA_{1c}: hemoglobin A_{1c}; HOMA2-β: homeostatic model assessment 2 estimates of β-cell function; HOMA2-IR: homeostatic model assessment 2 estimates of insulin resistance; HDL: high density lipoprotein; LDL: low density lipoprotein; eGFR: estimated glomerular filtration rate; CKD: chronic kidney disease.

ESM Table 2. Baseline characteristics of Ahlqvist's diabetes subtypes in Datasets B and C

Dataset B

Variables	All *n* =7,146	SAID *n* =495 (6.9%)	SIDD *n* =1,128 (15.8%)	SIRD *n* =965 (13.5%)	MOD *n* =2,077 (29.1%)	MARD *n* =2,418 (33.8%)	*p* value
Women, %	43.1	55.2	48.9	36.2	36.5	46.5	<0.001
Age, years	60 ± 14	53 ± 15	59 ± 13	56 ± 14	54 ± 13	70 ± 8	<0.001
(Min - Max)	(20 - 92)	(21 - 88)	(21-90)	(20-90)	(20-90)	(41 - 92)	<0.001
Age at diagnosis, years	50 ± 13	44 ± 14	47 ± 11	48 ± 12	40 ± 8	61 ± 8	<0.001
Diabetes duration, years	12 ± 10	10 ± 10	13 ± 11	9 ± 9	15 ± 12	10 ± 8	<0.001
Smoking, %	52.1	47.5	51.2	56.5	54.6	49.4	<0.001
Anthropometry							
BMI, kg/m^2	25.5 ± 5.5	22.9 ± 4.2	26.0 ± 4.9	30.2 ± 7.2	26.7 ± 5.5	23.1 ± 3.3	<0.001
Waist circumference, cm	91 ± 15	83 ± 11	93 ± 14	103 ± 18	94 ± 44	86 ± 14	<0.001
Systolic blood pressure, mmHg	129 ± 17	124 ±16	129 ± 18	131 ± 17	128 ± 16	130 ± 17	<0.001
Diastolic blood pressure, mmHg	76 ± 15	75 ± 35	76 ± 12	78 ± 12	77 ± 11	74 ± 11	<0.001
Biochemistry							
HbA1c							
%	7.5 ± 1.3	7.8 ± 1.5	9.6 ± 1.2	6.9 ± 0.9	7.0 ± 0.8	7.1 ± 0.7	<0.001
mmol/mol	58.3 ± 14.6	62.2 ± 16.8	81.2 ± 13.3	52.3 ± 13.3	52.9 ± 9.2	54.0 ± 8.1	<0.001
HOMA2-β	70.6 ± 47.9	43.7 ± 42.3	48.7 ± 25.0	146.8 ± 60.6	66.4 ± 34.1	59.8 ± 28.2	<0.001
HOMA2-IR	2.05 ± 1.61	1.01 ± 1.35	1.74 ± 1.08	4.77 ± 2.09	1.71 ± 0.91	1.61 ± 0.91	<0.001
Fasting plasma glucose							
mg/dL	136 ± 30	132 ± 34	152 ± 30	132 ± 31	133 ± 29	134 ± 26	<0.001
mmol/mol	7.6 ± 1.6	7.3 ± 1.9	8.4 ± 1.7	7.3 ± 1.7	7.4 ± 1.6	7.5 ± 1.5	<0.001
Triacylglycerol, mmol/l	1.69 ± 1.43	1.25 ± 0.88	1.95 ± 2.13	2.13 ± 1.55	1.70 ± 1.33	1.49 ± 1.04	<0.001
HDL cholesterol, mmol/l	1.42 ± 0.41	1.71 ± 0.52	1.37 ± 0.41	1.27 ± 0.34	1.40 ± 0.39	1.47 ± 0.41	<0.001
LDL cholesterol, mmol/l	2.74 ± 0.88	2.72 ± 0.80	2.92 ± 1.01	2.84 ± 0.93	2.69 ± 0.91	2.64 ± 0.75	<0.001
eGFR, ml/min per 1.73m^2	83 ± 20	86 ± 21	87 ± 22	84 ± 21	88 ± 22	78 ± 14	<0.001
Complications							
CKD G3a, %	0	0	0	0	0	0	
Proteinuria, %	12.9	8.7	19.7	15.9	12.4	10.0	<0.001
Hypertension, %	58.3	34.9	59.0	65.8	55.1	62.6	<0.001
Dyslipidemia, %	76.3	53.7	80.4	87.1	76.1	74.8	<0.001
Polyneuropathy, %	24.9	20.8	34.7	21.8	23.9	23.1	<0.001
Retinopathy, %	17.5	18.3	26	9.2	20.2	14.5	<0.001
Medications							
Metformin, %	50	18.6	57.1	45.5	51.8	38.5	<0.001
Insulin, %	34.6	80.6	56.6	14.9	31	25.9	<0.001

Dataset C

Variables	All *n* =5,844	SAID *n* =369 (6.3%)	SIDD *n* =771 (13.2%)	SIRD *n* =863 (14.8%)	MOD *n* =1,719 (29.4%)	MARD *n* =2,122 (36.3%)	*p* value
Women, %	43.9	55.3	51.2	37.8	38.0	46.4	<0.001
Age, years	63 ± 14	55 ± 15	62 ± 13	60 ± 14	56 ± 13	71 ± 8	<0.001
(Min - Max)	(20 - 97)	(21 -90)	(21 -93)	(20 - 93)	(21 - 89)	(39 - 97)	<0.001
Age at diagnosis, years	50 ± 13	46 ± 14	49 ± 12	51 ± 13	41 ± 8	61 ± 8	<0.001
Diabetes duration, years	11 ± 10	9 ± 9	12 ± 10	7 ± 8	14 ± 11	9 ± 7	<0.001
Smoking, %	49.8	44.2	49.2	54.6	52.3	46.7	<0.001
Anthropometry							
BMI, kg/m^2	25.3 ± 5.1	23.1 ± 4.2	25.8 ± 4.9	28.9 ± 6.3	26.4 ± 5.2	23.2 ± 3.2	<0.001
Waist circumference, cm	92 ± 34	82 ± 10	94 ± 14	99 ± 15	96 ± 60	86 ± 12	<0.001
Systolic blood pressure, mmHg	128 ± 20	124 ±17	130 ± 18	129 ± 17	127 ± 26	129 ± 16	<0.001
Diastolic blood pressure, mmHg	75 ± 19	75 ± 38	74 ± 12	77 ± 12	76 ± 25	73 ± 11	<0.001
Biochemistry							
HbA_{1c}							
%	7.4 ± 1.3	7.8 ± 1.5	9.5 ± 1.2	6.9 ± 1.0	6.9 ± 0.8	7.1 ± 0.7	<0.001
mmol/mol	56.9 ± 13.8	62.2 ± 16.2	80.3 ± 13.0	51.8 ± 10.5	52.1 ± 9.3	53.6 ± 8.1	<0.001
HOMA2-β	70.6 ± 47.9	43.7 ± 42.3	48.7 ± 25.0	146.8 ± 60.6	66.4 ± 34.1	59.8 ± 28.2	<0.001
HOMA2-IR	2.05 ± 1.61	1.01 ± 1.35	1.74 ± 1.08	4.77 ± 2.09	1.71 ± 0.91	1.61 ± 0.91	<0.001
Fasting plasma glucose							
mg/dL	135 ± 29	134 ± 34	153 ± 30	130 ± 30	131 ± 28	134 ± 26	<0.001
mmol/l	7.5 ± 1.6	8.5 ± 1.6	8.5 ± 1.6	7.2 ± 1.7	7.3 ± 1.6	7.4 ± 1.4	<0.001
Triacylglycerol, mmol/l	1.66 ± 1.35	1.31 ± 1.03	1.91 ± 1.84	2.07 ± 1.24	1.61 ± 1.15	1.51 ± 1.34	<0.001
HDL cholesterol, mmol/l	1.42 ± 0.41	1.73 ± 0.54	1.37 ± 0.39	1.29 ± 0.36	1.42 ± 0.39	1.47 ± 0.41	<0.001
LDL cholesterol, mmol/l	2.69 ± 0.85	2.72 ± 0.85	2.87 ± 0.96	2.77 ± 0.96	2.69 ± 0.83	2.59 ± 0.75	<0.001
eGFR, ml/min per 1.73m^2	76 ± 23	81 ± 24	80 ± 26	73 ± 24	82 ± 26	70 ± 18	<0.001
Complications							
CKD stage 3a, %	22.8	13.6	19.3	29.8	17.2	27.3	<0.001
Proteinuria, %	0	0	0	0	0	0	
Hypertension, %	62.9	38.2	64.2	70.3	59.4	66.4	<0.001
Dyslipidemia, %	78.8	58.3	83.1	88.3	78.1	77.6	<0.001
Polyneuropathy, %	27.9	23.9	41.7	24.8	25.5	26.7	<0.001
Retinopathy, %	17.6	20.3	26.5	10.1	20.9	14.6	<0.001
Medications							
Metformin, %	42	17.9	53.6	38.9	50.1	36.7	<0.001
Insulin, %	31.1	80.0	55.5	15.4	28.0	22.7	<0.001

Values are presented as mean ± SD, or n (%). P values were obtained by one-way ANOVA or Chi-square test. SAID: severe autoimmune diabetes; SIDD: severe insulin-deficient diabetes; SIRD: severe insulin-resistant diabetes; MOD: mild obesity-related diabetes; MARD: mild age-related diabetes; BMI: body mass index; HbA_{1c}: hemoglobin A_{1c}; HOMA2-β: homeostatic model assessment 2 estimates of β-cell function; HOMA2-IR: homeostatic model assessment 2 estimates of insulin resistance; HDL: high density lipoprotein; LDL: low density lipoprotein; eGFR: estimated glomerular filtration rate; CKD: chronic kidney disease.

ESM Table 3. Assessment of proportional hazards assumptions using Schoenfeld residuals tests in overall Cox regression models (Table 1, Model 5) for eGFR and proteinuria events

Variables	CKD stage 3a<br>Global p <0.001	CKD stage 3b<br>Global p =0.022	CKD stage 4<br>Global p =0.254	CKD stage 5<br>Global p =0.355	Proteinuria<br>Global p =0.023
SAID	0.131	0.104	0.175	0.050	0.075
SIDD	0.141	0.343	0.772	0.156	0.980
SIRD	0.431	0.394	0.775	0.678	0.326
MOD	0.440	0.958	0.085	0.253	0.303
MARD	-	-	-	-	-
Age per year	0.051	0.006	0.043	0.259	0.084
Men vs women	0.102	0.335	0.915	0.815	0.199
Diabetes duration per year	0.097	0.769	0.1	0.156	0.237
BMI per kg/m^2	0.905	0.065	0.162	0.798	0.912
HbA_{1c} per mmol/mol	0.005	0.614	0.679	0.519	0.795
Current or ex-smoking vs never smoking	0.687	0.038	0.581	0.716	0.305
Hypertension yes vs no	0.432	0.061	0.495	0.134	0.207
Dyslipidemia yes vs no	0.009	0.991	0.469	0.792	0.076
eGFR, mL/min per 1.73 m^2	<0.001	0.348	0.414	0.413	0.001

The Grambsch-Therneau test indicates a violation of the proportional hazards assumption when $p < 0.05$. Global tests assess the overall model assumption. Individual tests evaluate each covariate separately, with the MARD subtype serving as the reference category. All models correspond to Table 1, Model 5 (a fully adjusted model). SAID: severe autoimmune diabetes; SIDD: severe insulin-deficient diabetes; SIRD: severe insulin-resistant diabetes; MOD: mild obesity-related diabetes; MARD: mild age-related diabetes; BMI: body mass index; HbA_{1c}: hemoglobin A_{1c}; eGFR: estimated glomerular filtration rate; CKD: chronic kidney disease.

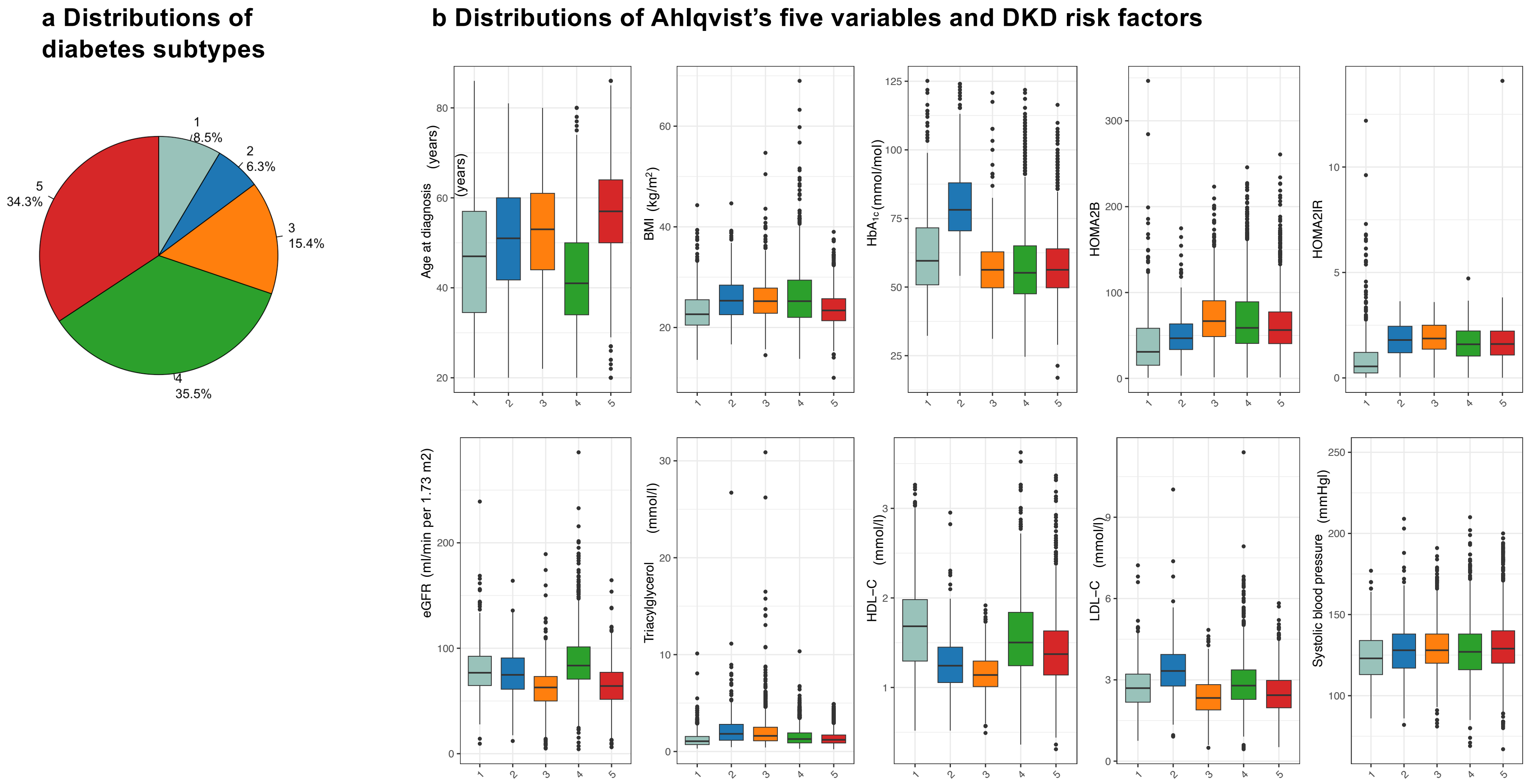

ESM Fig. 6 (**a**) Distributions (%) of diabetes subtypes in J-DREAMS using Ahlqvist’s five variables plus five diabetic kidney disease (DKD) risk factors. Numbers are cluster 1, cluster 2, cluster 3, cluster 4, and cluster 5. Ahlqvist's variables include age at diagnosis, BMI, HbA_{1c}, HOMA2-β, HOMA2-IR and DKD risk factors include eGFR, triacylglycerol ,HDL cholesterol, LDL cholesterol, and systolic blood pressure. (**b**) Distributions of Ahlqvist’s five variables and DKD risk factors in clusters 1-5 as in (a). BMI: body mass index; HbA_{1c}: hemoglobin A_{1c}; HOMA2-β, : homeostatic model assessment 2 estimates of β-cell function; HOMA2-IR: homeostatic model assessment 2 estimates of insulin resistance; HDL: high density lipoprotein; LDL: low density lipoprotein; eGFR: estimated glomerular filtration rate. Whiskers show median with interquartile range (IQR) and bars indicate 95% confidence interval (CI).

ESM Table 4. Baseline characteristics of alternative diabetes subtypes determined by using Ahlqvist's variables plus classical DKD risk factors

Variables	Cluster 1 *n* = 447(8.5%)	Cluster 2 *n* =329 (6.3%)	Cluster 3 *n* =805 (15.4%)	Cluster 4 *n* =1,856(35.5%)	Cluster 5 *n* =1,793 (34.3%)	*p* value
Women, %	55.7	48.7	20.9	36.3	43.4	<0.001
Age, years	56 ± 15	63 ± 13	58 ± 13	56 ± 14	72 ± 8	<0.001
Age at diagnosis, years	46 ± 14	51 ± 12	53 ± 12	43 ± 11	57 ± 11	<0.001
Diabetes duration,years	10 ± 9	14 ± 11	9 ± 9	16 ± 12	11 ± 8	<0.001
Smoking, %	44.2	50.9	53.2	53.6	48.5	<0.001
Anthropometry						
BMI, kg/m^2	23.4 ± 4.3	25.8 ± 4.4	25.7 ± 4.2	26.1 ± 5.8	23.6 ± 3.4	<0.001
Systolic blood pressure, mmHg	124 ± 16	129 ± 17	129 ± 16	128 ± 17	130 ± 27	<0.001
Diastolic blood pressure, mmHg	75 ± 35	79 ± 13	79 ± 12	76 ± 25	72 ± 12	<0.001
Biochemistry						
HbA_{1c}						
%	7.9 ± 1.5	9.6 ± 1.3	7.3 ± 1.0	7.4 ± 1.4	7.4 ± 1.1	<0.001
mmol/mol	63 ± 17	82 ± 13	51 ± 10	53 ± 10	55 ± 8	<0.001
HOMA2-β	42.4 ± 39.9	51.1 ± 25.8	72.3 ± 33.2	68.1 ± 38.3	62.3 ± 32.5	<0.001
HOMA2-IR	1.04 ± 1.40	1.82 ± 0.84	1.90 ± 0.77	1.63 ± 0.81	1.68 ± 0.85	<0.001
Fasting plasma glucose						
mg/dL	133 ± 34	150 ± 34	134 ± 26	107 ± 21	129 ± 29	<0.001
mmol/l	7.4 ± 1.9	8.3 ± 1.9	7.4 ± 1.4	5.9 ± 1.2	7.2 ± 1.6	<0.001
Triacylglycerol, mmol/l	1.33 ± 0.99	2.28 ± 2.03	2.27 ± 2.30	1.54 ± 0.94	1.39 ± 0.72	<0.001
HDL cholesterol, mmol/l	1.71 ± 0.54	1.27 ± 0.34	1.14 ± 0.23	1.58 ± 0.44	1.42 ± 0.39	<0.001
LDL cholesterol, mmol/l	2.72 ± 0.85	3.36 ± 0.98	2.38 ± 0.67	2.87 ± 0.91	2.48 ± 0.72	<0.001
eGFR, ml/min per $1.73m^2$	80 ± 25	76 ± 25	71 ± 25	78 ± 28	68 ± 19	<0.001

Values are presented as mean ± SD, or n (%). *P* values were obtained by one-way ANOVA test or Chi-square test. DKD: diabetic kidney disease; BMI: body mass index; HbA_{1c}: hemoglobin A_{1c}; HOMA2-β: homeostatic model assessment 2 estimates of β-cell function; HOMA2-IR: homeostatic model assessment 2 estimates of insulin resistance; HDL: high density lipoprotein; LDL: low density lipoprotein; eGFR: estimated glomerular filtration rate.

ESM Table 5. Overall Cox proportional hazards model for eGFR and proteinuria events using alternative diabetes subtypes determined by Ahlqvist's variables plus classical DKD risk factors

CKD stage 3a

Variables	Model HR (95%CI)	*p* value
Cluster1	1.13 (0.99 - 1.43)	0.281
Cluster2	1.43 (1.12 - 1.82)	0.004
Cluster3	1.28 (1.08 - 1.52)	0.004
Cluster4	0.71 (0.61 - 0.84)	<0.001
Cluster5	1.00 (ref)	-
Age per year	1.04 (1.03 - 1.04)	<0.001
Men vs women	1.08 (0.96 - 1.22)	0.216
Diabetes duration per year	1.00 1(1.00 - 1.01)	0.146

CKD stage 3b

Variables	Model HR (95%CI)	*p* value
Cluster1	1.18 (0.71 - 1.95)	0.529
Cluster2	1.60 (0.94 - 2.70)	0.083
Cluster3	1.34 (0.92 - 1.06)	0.123
Cluster4	0.93 (0.70 - 1.32)	0.703
Cluster5	1.00 (ref)	-
Age per year	1.02 (1.01 - 1.04)	0.001
Men vs women	1.16 (0.89 - 1.52)	0.265
Diabetes duration per year	1.02 (1.00 - 1.03)	0.017

CKD stage 4

Variables	Model HR (95%CI)	*p* value
Cluster1	0.75 (0.24- 2.34)	0.624
Cluster2	1.72 (0.62 - 4.72)	0.295
Cluster3	1.12 (0.51 - 2.43)	0.778
Cluster4	1.02 (0.51 - 2.02)	0.958
Cluster5	1.00 (ref)	-
Age per year	1.02 (1.00 - 1.05)	0.081
Men vs women	0.92 (0.55- 1.56)	0.756
Diabetes duration per year	1.00 (0.97 - 1.03)	0.927

CKD stage 5

Variables	Model HR (95%CI)	*p* value
Cluster1	not detected	–
Cluster2	3.17 (0.48 - 20.82)	0.230
Cluster3	3.17 (0.07 - 6.27)	0.708
Cluster4	0.81 (0.15 - 4.37)	0.805
Cluster5	1.00 (ref)	-
Age per year	0.99 (0.93 - 1.06)	0.832
Men vs women	1.00 (0.28 - 3.57)	0.998
Diabetes duration per year	1.01 (0.94 - 1.09)	0.789

DKD: diabetic kidney disease; BMI: body mass index; HbA_{1c}: Hemoglobin A_{1c}; eGFR: estimated glomerular filtration rate; CKD: chronic kidney disease.

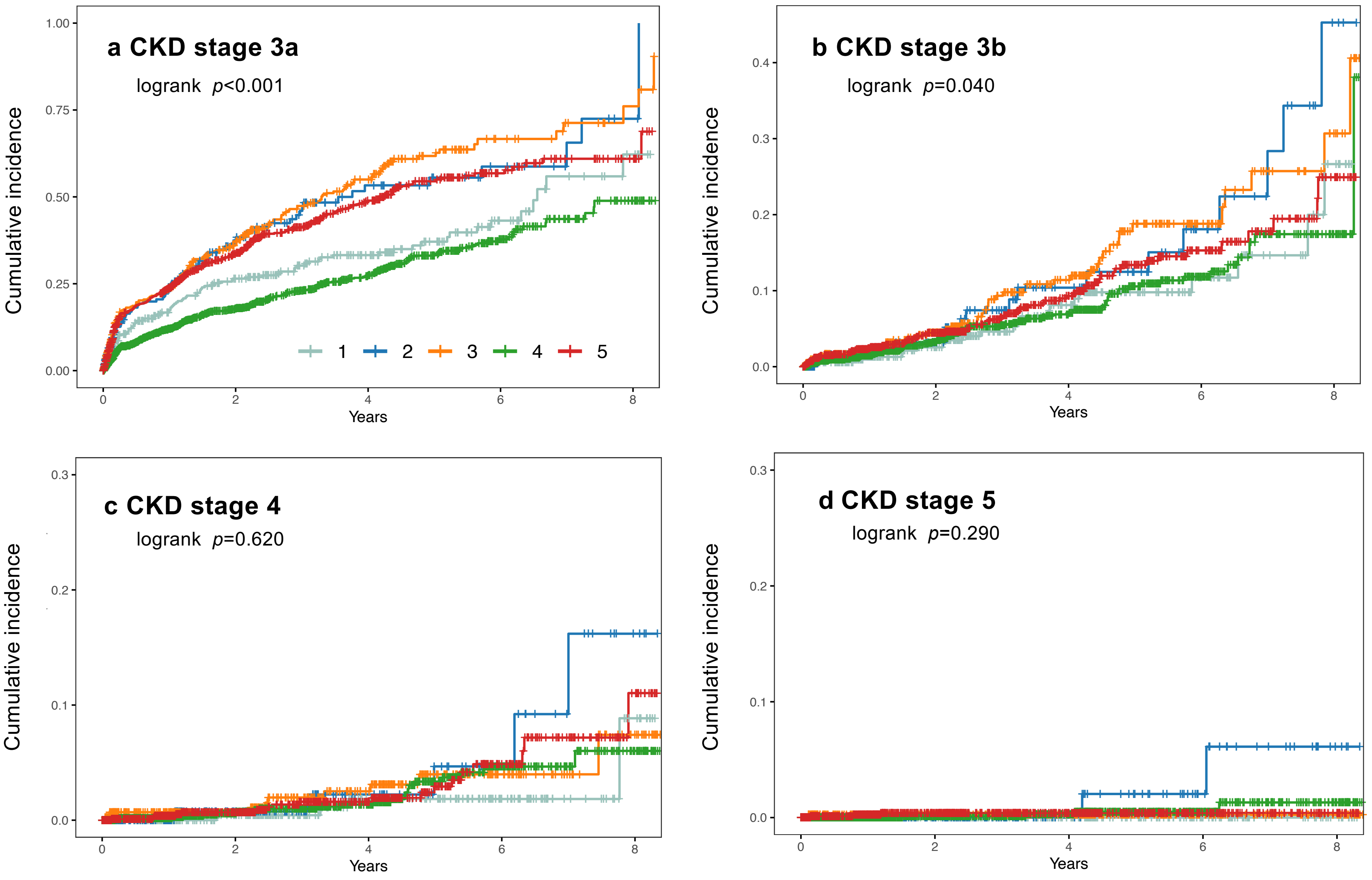

ESM Fig.7 Kaplan-Meier curves for the development of (**a**) stage 3a (eGFR <60 mL/min/1.73 m^2), (**b**) stage 3 (eGFR <45 mL/min/1.73 m^2), (**c**) stage 4 (eGFR <30 mL/min/1.73 m^2), and (**d**) stage 5 (eGFR <15 mL/min/1.73 m^2) in individuals with cluster 1 (light blue lines), cluster 2 (blue lines), cluster 3 (orange lines), cluster 4 (green lines), and cluster 5 (red lines). Diabetes subtypes were determined using Ahlqvist's five variables plus five diabetic kidney disease (DKD) in Dataset D (n=5,220). Ahlqvist's variables include age at diagnosis, BMI, HbA_{1c}, HOMA2-β, HOMA2-IR and DKD risk factors include eGFR, systolic blood pressure, HDL cholesterol, LDL cholesterol, triacylglycerol. eGFR: estimated glomerular filtration rate. Logrank p values are shown.

ESM Table 6. Sub-analysis in overall Cox proportional hazards model (Model 5, Table 1) for eGFR and proteinuria events

CKD stage G3b

	Age						Sex					
	< 65			65 ≤			Men			Women		
Variables	HR (95%CI)	p value	*q* value	HR (95%CI)	p value	*q* value	HR (95%CI)	p value	*q* value	HR (95%CI)	p value	*q* value
SAID	0.35 (0.16 - 0.75)	0.007	0.091	1.26 (0.85 - 1.86)	0.239	0.345	0.92 (0.55 - 1.53)	0.735	0.802	1.15 (0.73 - 1.85)	0.535	0.769
SIDD	0.91 (0.47 - 1.76)	0.773	0.973	1.32(0.99 - 1.77)	0.058	0.108	1.35 (0.95 - 1.90)	0.090	0.155	1.49 (0.98 - 2.25)	0.056	0.183
SIRD	0.64 (0.39 - 1.05)	0.080	0.208	1.31 (1.08 - 1.75)	0.005	0.016	1.24 (1.00 - 1.53)	0.050	0.150	1.23 (0.92 - 1.64)	0.156	0.374
MOD	0.56 (0.35 - 0.91)	0.018	0.117	1.45 (1.14 - 1.84)	0.002	0.009	1.24 (0.97 - 1.59)	0.082	0.155	1.42 (0.99 - 2.03)	0.060	0.183
MARD	1.00 (ref)	-		1.00 (ref)	-		1.00 (ref)	-		1.00 (ref)	-	-
Age per year	1.00 (0.99 - 1.03)	0.474	0.943	1.02 (1.01 - 1.03)	0.001	0.007	1.02 (1.00 - 1.03)	<0.001	0.012	1.01 (1.00 - 1.02)	0.269	0.461
Men vs women	1.05 (0.79 - 1.40)	0.726	0.973	1.06 (0.90 - 1.24)	0.495	0.644	-	-		-	-	-
Diabetes duration per year	1.00 (0.99 - 1.02)	0.908	0.984	0.99 (0.98 - 0.99)	<0.001	0.007	0.99 (0.98 - 1.00)	0.039	0.150	0.98 (0.97 - 1.00)	0.061	0.183
BMI per kg/m^2	1.02 (1.00 - 1.04)	0.071	0.208	0.99 (0.97 - 1.01)	0.238	0.345	1.01 (1.00 - 1.03)	0.116	0.155	0.98 (0.96 - 1.00)	0.004	0.048
HbA_{1c} per mmol/mol	1.00 (0.99 - 1.01)	0.508	0.943	1.01 (1.00 - 1.02)	0.009	0.023	1.01 (1.00 - 1.01)	0.106	0.155	1.01 (1.00 - 1.01)	0.257	0.461
Current or ex-smoking vs never smoking	1.02 (0.86 - 1.20)	0.823	0.973	1.02 (0.94 - 1.11)	0.615	0.666	0.99 (0.91 - 1.09)	0.908	0.908	1.01 (0.88 - 1.17)	0.842	0.842
Hypertension yes vs no	1.00 (0.70 - 1.42)	0.989	0.989	0.97 (0.79 - 1.18)	0.746	0.746	0.96 (0.78 - 1.18)	0.106	0.155	0.92 (0.67 - 1.25)	0.577	0.769
Dyslipidemia yes vs no	1.41 (0.99 - 1.20)	0.060	0.208	0.95 (0.79 - 1.15)	0.606	0.666	0.95 (0.77 - 1.17)	0.621	0.745	1.04 (0.79 - 1.37)	0.780	0.842
eGFR, mL/min per 1.73 m^2	1.00 (0.99 - 1.01)	0.690	0.973	0.99 (0.98 - 1.00)	0.058	0.108	0.99 (0.98 - 1.00)	0.015	0.090	1.00 (0.99 - 1.01)	0.654	0.785

	Diabetes duration								
	< 2 years			2-10 years			≥ 10 years		
Variables	HR (95%CI)	p value	*q* value	HR (95%CI)	p value	*q* value	HR (95%CI)	p value	*q* value
SAID	1.53 (0.42 - 5.54)	0.519	0.911	0.87 (0.44 - 1.74)	0.706	0.834	0.99 (0.65 - 1.52)	0.967	0.967
SIDD	1.47 (0.40 - 5.38)	0.561	0.911	1.63 (0.75 - 3.55)	0.219	0.569	1.18 (0.88 - 1.58)	0.273	0.507
SIRD	1.91 (0.96 - 3.80)	0.067	0.497	1.55 (1.11 - 2.13)	0.009	0.117	1.06 (0.85 - 1.31)	0.618	0.805
MOD	0.78 (0.15 - 4.03)	0.772	0.911	1.24 (0.73 - 2.11)	0.422	0.686	1.15 (0.92 - 1.44)	0.218	0.472
MARD	1.00 (ref)	-	-	1.00 (ref)	-	-	1.00 (ref)	-	-
Age per year	1.02 (0.99 - 1.05)	0.115	0.497	1.01 (1.00 - 1.03)	0.105	0.455	1.01 (1.00 - 1.02)	0.021	0.091
Men vs women	1.12 (0.53 - 2.36)	0.763	0.911	1.07 (0.80 - 1.43)	0.647	0.834	1.03 (0.87 - 1.21)	0.763	0.827
Diabetes duration per year	1.02 (0.54 - 1.92)	0.955	0.955	0.95 (0.90 - 1.01)	0.075	0.455	0.99 (0.98 - 1.00)	0.009	0.059
BMI per kg/m^2	0.93 (0.86 - 1.01)	0.079	0.497	1.02 (0.98 - 1.05)	0.339	0.635	1.00 (0.98 - 1.01)	0.681	0.805
HbA_{1c} per mmol/mol	1.01 (0.99 - 1.04)	0.323	0.840	0.99 (0.98 - 1.01)	0.342	0.635	1.01 (1.00 - 1.02)	0.002	0.026
Current or ex-smoking vs never smoking	1.13 (0.75 - 1.69)	0.562	0.911	0.90 (0.76 - 1.06)	0.205	0.569	1.06 (0.97 - 1.16)	0.171	0.445
Hypertension yes vs no	1.89 (0.79 - 4.54)	0.153	0.497	1.12 (0.77 - 1.62)	0.542	0.783	0.90 (0.73 - 1.11)	0.341	0.554
Dyslipidemia yes vs no	1.08 (0.53 - 2.20)	0.841	0.911	1.00 (0.67 - 1.50)	0.988	0.988	1.46 (0.87 - 1.26)	0.638	0.805
eGFR, mL/min per 1.73 m^2	1.01 (0.97 - 1.04)	0.754	0.911	1.00 (0.99 - 1.02)	0.963	0.988	0.99 (0.99 - 1.00)	0.033	0.107

BMI												HbA1c					
< 18.5			18.5 - 22			22 - 25			25 ≤			< 63			≥ 63		
HR (95%CI)	p value	*q* value	HR (95%CI)	p value	*q* value	HR (95%CI)	p value	*q* value	HR (95%CI)	p value	*q* value	HR (95%CI)	p value	*q* value	HR (95%CI)	p value	*q* value
0.63 (0.34 - 1.21)	0.169	0.439	1.08 (0.55 - 2.12)	0.828	0.955	1.16 (0.65 - 2.06)	0.621	0.767	1.02 (0.58 - 1.81)	0.935	0.967	0.95 (0.63 - 1.45)	0.830	0.870	1.14 (0.62 - 2.07)	0.678	0.802
1.00 (0.54 - 1.84)	0.995	0.995	1.32 (0.67 - 2.61)	0.427	0.925	1.30 (0.82 - 2.06)	0.263	0.570	1.86 (1.25 - 2.88)	0.002	0.026	4.04 (0.56 - 29.32)	0.168	0.394	1.24 (0.85 - 1.80)	0.262	0.626
1.92 (1.05 - 3.54)	0.035	0.228	1.02 (0.67 - 1.55)	0.930	0.955	1.37 (0.99 - 1.90)	0.060	0.156	1.44 (1.11 - 1.88)	0.007	0.046	1.21 (1.00 - 1.46)	0.054	0.234	1.24 (0.83 - 1.84)	0.289	0.626
0.93 (0.57 - 1.52)	0.782	0.936	0.99 (0.61 - 1.58)	0.955	0.955	1.18 (0.80 - 1.74)	0.400	0.650	1.46 (1.08 - 1.97)	0.014	0.061	1.18 (0.95 - 1.48)	0.142	0.394	1.24 (0.79 - 1.96)	0.342	0.635
1.00 (ref)	-	-	1.00 (ref)	-	-	1.00 (ref)	-	-	1.00 (ref)	-	-	1.00 (ref)	-	-	1.00 (ref)	-	-
1.00 (0.99 - 1.02)	0.717	0.936	1.02 (1.01 - 1.04)	0.004	0.052	1.02 (1.00 - 1.03)	0.028	0.121	1.01 (1.00 - 1.02)	0.160	0.517	1.01 (1.00 - 1.02)	0.012	0.104	1.01 (1.00 - 1.03)	0.067	0.290
1.11 (0.78 - 1.57)	0.566	0.920	0.93 (0.68 - 1.28)	0.660	0.955	1.12 (0.83 - 1.51)	0.461	0.666	1.05 (0.86 - 1.28)	0.644	0.892	1.04 (0.89 - 1.23)	0.601	0.870	0.99 (0.77 - 1.29)	0.957	0.957
1.01 (0.99 - 1.02)	0.305	0.661	1.00 (0.98 - 1.01)	0.596	0.955	0.98 (0.97 - 0.99)	0.001	0.013	0.99 (0.98 - 1.00)	0.199	0.517	0.99 (0.98 - 1.00)	0.016	0.104	0.99 (0.97 - 1.00)	0.014	0.182
1.01 (0.87 - 1.16)	0.938	0.995	0.90 (0.79 1.03)	0.142	0.637	0.98 (0.86 - 1.11)	0.708	0.767	1.00 (0.97 - 1.02)	0.757	0.895	1.01 (0.99 - 1.02)	0.317	0.589	0.97 (0.95 - 1.00)	0.053	0.290
1.00 (0.99 - 1.01)	0.792	0.936	1.00 (0.99 - 1.02)	0.763	0.955	1.01 (1.00 - 1.02)	0.045	0.146	1.00 (0.99 - 1.00)	0.378	0.702	1.00 (0.99 - 1.01)	0.811	0.870	1.00 (0.99 - 1.01)	0.740	0.802
0.93 (0.75 - 1.16)	0.542	0.920	0.89 (0.74 - 1.06)	0.200	0.637	1.03 (0.89 - 1.19)	0.690	0.767	1.07 (0.96 - 1.20)	0.241	0.522	0.99 (0.91 - 1.08)	0.870	0.870	1.09 (0.94 - 1.26)	0.267	0.626
1.37 (0.96 - 1.96)	0.081	0.351	0.82 (0.58 - 1.15)	0.245	0.637	0.98 (0.71 - 1.36)	0.902	0.902	1.01 (0.75 - 1.35)	0.967	0.967	0.98 (0.80 - 1.20)	0.827	0.870	1.00 (0.67 - 1.01)	0.610	0.802
1.31 (0.92 - 1.85)	0.131	0.426	1.05 (0.77 - 1.44)	0.740	0.955	0.87 (0.65 - 1.17)	0.364	0.650	0.94 (0.69 - 1.27)	0.686	0.892	1.02 (0.91 - 1.09)	0.823	0.870	0.92 (0.63 - 1.34)	0.679	0.802
0.95 (0.95 - 0.96)	<0.001	0.013	0.99 (0.98 - 1.01)	0.203	0.637	0.99 (0.98 - 1.00)	0.025	0.121	1.00 (0.99 - 1.01)	0.680	0.892	1.00 (0.99 - 1.00)	0.182	0.394	1.00 (0.98 - 1.01)	0.516	0.802

Proteinuria

	Age						Sex					
	< 65			65 ≤			Men			Women		
Variables	HR (95%CI)	p value	*q* value	HR (95%CI)	p value	*q* value	HR (95%CI)	p value	*q* value	HR (95%CI)	p value	*q* value
SAID	1.03 (0.59 - 1.79)	0.911	0.911	0.70 (0.49 - 1.03)	0.067	0.145	1.17 (0.84 - 1.62)	0.344	0.523	0.83 (0.61 - 1.14)	0.247	0.598
SIDD	0.95 (0.64 - 1.43)	0.821	0.889	0.80 (0.62 - 1.03)	0.079	0.147	0.90 (0.71 - 1.13)	0.351	0.523	0.89 (0.69 - 1.16)	0.393	0.674
SIRD	1.12 (0.81 - 1.54)	0.497	0.718	0.90 (0.75 - 1.08)	0.251	0.363	1.01 (0.87 - 1.18)	0.855	0.933	0.99 (0.80 - 1.24)	0.958	0.962
MOD	1.12 (0.83 - 1.51)	0.449	0.718	1.03 (0.83 - 1.28)	0.799	0.799	1.00 (0.85 - 1.17)	0.986	0.986	0.86 (0.68 - 1.09)	0.200	0.598
MARD	1.00 (ref)	-	-	1.00 (ref)	-	-	1.00 (ref)	-	-	1.00 (ref)	-	-
Age per year	1.91 (0.99 - 1.02)	0.312	0.579	1.03 (1.02 - 1.04)	<0.001	0.004	1.01 (1.00 - 1.02)	<0.001	0.003	1.00 (0.99 - 1.01)	0.877	0.962
Men vs women	1.07 (0.85 - 1.35)	0.116	0.503	1.03 (0.89 - 1.19)	0.733	0.794	-	-	-	-	-	-
Diabetes duration per year	1.01 (1.00 - 1.02)	0.561	0.729	1.00 (0.99 - 1.00)	0.352	0.458	1.00 (0.99 - 1.00)	0.136	0.326	1.00 (0.99 - 1.00)	0.254	0.598
BMI per kg/m^2	0.99 (0.97 - 1.01)	0.216	0.579	1.02 (1.01 - 1.04)	0.003	0.010	1.00 (0.98 - 1.01)	0.603	0.724	1.00 (0.98 - 1.01)	0.948	0.962
HbA_{1c} per mmol/mol	1.00 (0.99 - 1.01)	0.727	0.859	1.01 (1.00 - 1.01)	0.001	0.004	1.01 (1.01 - 1.02)	<0.001	0.003	1.00 (1.00 - 1.01)	0.299	0.598
Current or ex-smoking vs never smoking	0.89 (0.72 - 1.11)	0.297	0.579	0.87 (0.76 - 1.00)	0.053	0.138	0.96 (0.95 - 0.96)	0.392	0.523	1.00 (0.84 - 1.18)	0.962	0.962
Hypertension yes vs no	1.57 (1.19 - 2.08)	0.001	0.007	1.13 (0.94 - 1.37)	0.186	0.302	1.31 (1.14 - 1.50)	<0.001	0.003	1.03 (0.85 - 1.26)	0.734	0.962
Dyslipidemia yes vs no	1.18 (0.87 - 1.60)	0.278	0.579	1.04 (0.87 - 1.25)	0.636	0.752	1.07 (0.93 - 1.24)	0.318	0.523	1.16 (0.95 - 1.41)	0.142	0.598
eGFR, mL/min per 1.73 m^2	0.93 (0.92 - 0.93)	<0.001	0.001	0.91 (0.91 - 0.91)	<0.001	0.004	0.96 (0.95 - 0.96)	<0.001	0.003	0.95 (0.95 - 0.96)	<0.001	0.012

	Diabetes duration								
	< 2 years			2-10 years			≥ 10 years		
Variables	HR (95%CI)	p value	*q* value	HR (95%CI)	p value	*q* value	HR (95%CI)	p value	*q* value
SAID	1.01 (0.45 - 2.77)	0.814	0.962	1.00 (0.57 - 1.78)	0.990	0.993	0.78 (0.52 - 1.15)	0.205	0.267
SIDD	1.00 (0.49 - 2.03)	0.996	0.996	0.98 (0.57 - 1.69)	0.939	0.993	0.78 (0.61 - 1.01)	0.056	0.134
SIRD	1.31 (0.77 - 2.22)	0.316	0.587	1.26 (0.96 - 1.66)	0.099	0.429	0.85 (0.69 - 1.03)	0.101	0.188
MOD	0.90 (0.42 - 1.93)	0.796	0.962	1.36 (0.90 - 2.04)	0.140	0.455	1.00 (0.82 - 1.21)	0.972	0.972
MARD	1.00 (ref)	-	-	1.00 (ref)	-	-	1.00 (ref)	-	-
Age per year	1.02 (1.00 - 1.04)	0.013	0.085	1.02 (1.00 - 1.03)	0.005	0.033	1.02 (1.01 - 1.03)	<0.001	0.004
Men vs women	1.58 (1.00 - 2.52)	0.052	0.169	1.02 (0.80 - 1.03)	0.850	0.993	1.02 (0.88 - 1.19)	0.782	0.847
Diabetes duration per year	0.87 (0.56 - 1.35)	0.521	0.847	1.01 (0.96 - 1.06)	0.766	0.993	0.99 (0.99 - 1.00)	0.126	0.205
BMI per kg/m^2	0.97 (0.93 - 1.02)	0.229	0.496	1.01 (0.99 - 1.03)	0.450	0.902	1.01 (1.00 - 1.02)	0.152	0.220
HbA_{1c} per mmol/mol	1.00 (0.99 - 1.02)	0.988	0.996	1.00 (0.99 - 1.01)	0.993	0.993	1.01 (1.00 - 1.02)	0.001	0.004
Current or ex-smoking vs never smoking	0.60 (0.38 - 0.95)	0.030	0.130	0.93 (0.73 - 1.18)	0.555	0.902	0.87 (0.76 - 1.01)	0.062	0.134
Hypertension yes vs no	1.45 (0.89 - 2.35)	0.136	0.354	1.22 (0.89 - 1.66)	0.210	0.546	1.24 (1.02 - 1.51)	0.028	0.091
Dyslipidemia yes vs no	0.90 (0.54 - 1.49)	0.681	0.962	1.12 (0.79 - 1.59)	0.535	0.902	1.09 (0.91 - 1.30)	0.374	0.442
eGFR, mL/min per 1.73 m^2	0.93 (0.92 - 0.94)	<0.001	0.013	0.91 (0.91 - 0.92)	<0.001	0.013	0.92 (0.91 - 0.92)	<0.001	0.004

BMI												HbA1c					
< 18.5			18.5 - 22			22 - 25			25 ≤			< 63			≥ 63		
HR (95%CI)	P value	q value	HR (95%CI)	p value	*q* value	HR (95%CI)	p value	*q* value	HR (95%CI)	p value	*q* value	HR (95%CI)	p value	*q* value	HR (95%CI)	p value	*q* value
0.32 (0.06 - 1.87)	0.206	0.586	1.07 (0.61 - 1.67)	0.823	0.962	0.77 (0.46 - 1.30)	0.330	0.715	0.96 (0.57 - 1.63)	0.892	0.950	1.05 (0.73 - 1.52)	0.781	0.818	0.71 (0.42 - 1.21)	0.211	0.458
0.64 (0.19 - 2.17)	0.476	0.586	0.95 (0.55 - 1.64)	0.857	0.962	0.90 (0.61 - 1.31)	0.571	0.823	0.80 (0.58 - 1.09)	0.150	0.325	0.25 (0.08 - 0.80)	0.019	0.049	0.95 (0.70 - 1.29)	0.730	0.730
0.66 (0.29 - 1.49)	0.316	0.586	0.82 (0.54 - 1.21)	0.313	0.814	1.10 (0.82 - 1.47)	0.525	0.823	1.01 (0.81 - 1.26)	0.950	0.950	0.96 (0.81 - 1.14)	0.669	0.791	1.08 (0.76 - 1.54)	0.653	0.707
0.75 (0.33 - 1.71)	0.496	0.586	1.00 (0.66 - 1.50)	0.995	0.995	1.07 (0.77 - 1.47)	0.696	0.823	1.08 (0.84 - 1.37)	0.557	0.658	1.04 (0.87 - 1.26)	0.659	0.791	1.12 (0.76 - 1.66)	0.571	0.675
1.00 (ref)	-	-	1.00 (ref)	-	-	1.00 (ref)	-	-	1.00 (ref)	-	-	1.00 (ref)	-	-	1.00 (ref)	-	-
1.00 (0.97 - 1.02)	0.852	0.923	1.02 (1.00 - 1.03)	0.090	0.390	1.02 (1.01 - 1.03)	0.001	0.007	1.02 (1.01 - 1.03)	<0.001	0.007	1.02 (1.01 - 1.02)	<0.001	0.007	1.02 (1.01 - 1.03)	<0.001	0.007
1.39 (0.80 - 2.42)	0.244	0.586	1.02 (0.76 - 1.38)	0.873	0.962	0.89 (0.68 - 1.11)	0.255	0.663	1.09 (0.91 - 1.30)	0.356	0.514	0.98 (0.85 - 1.14)	0.818	0.818	1.13 (0.90 - 1.43)	0.288	0.458
1.01 (0.99 - 1.04)	0.272	0.586	1.00 (0.98 - 1.01)	0.620	0.962	1.00 (0.99 - 1.01)	0.418	0.776	1.00 (1.00 - 1.01)	0.287	0.497	1.00 (0.99 - 1.00)	0.471	0.703	1.01 (1.00 - 1.01)	0.231	0.458
1.10 (0.87 - 1.39)	0.419	0.586	1.08 (0.94 - 1.23)	0.284	0.814	1.03 (0.91 - 1.16)	0.665	0.823	0.99 (0.97 - 1.01)	0.457	0.594	1.01 (0.99 - 1.02)	0.414	0.703	1.01 (0.99 - 1.03)	0.323	0.458
1.01 (0.99 - 1.04)	0.336	0.586	1.00 (0.99 - 1.01)	0.629	0.962	1.01 (1.00 - 1.02)	0.007	0.030	1.01 (1.00 - 1.01)	0.132	0.325	1.01 (1.00 - 1.02)	0.013	0.042	1.01 (0.99 - 1.02)	0.352	0.458
0.62 (0.36 - 1.08)	0.091	0.586	1.03 (0.79 - 1.35)	0.831	0.962	0.98 (0.78 - 1.22)	0.825	0.825	0.79 (0.64 - 0.92)	0.002	0.009	0.86 (0.75 - 0.99)	0.036	0.078	0.92 (0.74 - 1.15)	0.352	0.458
1.03 (0.55 - 1.91)	0.936	0.936	1.32 (0.97 - 1.80)	0.082	0.390	1.39 (1.04 - 1.86)	0.026	0.085	1.21 (0.94 - 1.55)	0.146	0.325	1.32 (1.10 - 1.59)	0.003	0.013	1.18 (0.89 - 1.58)	0.250	0.458
1.30 (0.73 - 2.33)	0.378	0.586	0.98 (0.79 - 1.35)	0.888	0.962	0.97 (1.00 - 1.03)	0.805	0.825	1.15 (0.88 - 1.51)	0.306	0.497	1.06 (0.86 - 1.26)	0.487	0.703	1.19 (0.84 - 1.67)	0.324	0.458
0.92 (0.91- 0.93)	<0.001	0.013	0.92 (0.91 - 0.92)	<0.001	0.013	0.91 (0.91 - 0.92)	<0.001	0.007	0.92 (0.91 - 0.92)	<0.001	0.007	0.92 (0.91 - 0.92)	<0.001	0.007	0.92 (0.91 - 0.92)	<0.001	0.007

SAID: severe autoimmune diabetes; SIDD: severe insulin-deficient diabetes; SIRD: severe insulin-resistant diabetes; MOD: mild obesity-related diabetes; MARD: mild age-related diabetes; BMI: body mass index; HbA1c: Hemoglobin A1c; eGFR: estimated glomerular filtration rate; CKD: chronic kidney disease.

ESM Table 7. Baseline characteristics of diabetes subtypes with and without eGFR and proteinuria events

CKD stage 3b (Dataset B)

	SAID			SIDD			SIRD			MOD			MARD		
	Stage 3b			Stage 3b			Stage 3b			Stage 3b			Stage 3b		
Variables	Event	No event	p value	Event	No event	p value	Event	No event	p value	Event	No event	p value	Event	No event	p value
Age, years	63 ± 11	56 ± 16	0.029	66 ± 11	62 ± 13	0.967	61 ± 12	59 ± 15	0.245	62 ± 11	55 ± 14	<0.001	69.6 ± 6.8	71.3 ± 8.1	0.016
Women, %	62.5	54.5	0.574	52.5	47.5	0.532	25.3	34.9	0.138	29.9	35.5	0.251	39.0	44.7	0.217
Diabetes duration, years	13.0 ± 10.8	9.7 ± 9.6	0.099	15.2 ± 10.6	13.5 ± 11.0	0.228	11.3 ± 7.7	8.8 ± 8.8	0.019	20.3 ± 12.3	15.1 ± 12.1	<0.001	9.8 ± 7.0	10.1 ± 7.8	0.574
BMI, kg/m^2	23.7 ± 5.2	23.0 ± 4.1	0.444	26.9 ± 5.4	25.9 ± 4.8	0.106	28.1 ± 5.9	29.0 ± 5.3	0.917	26.4 ± 5.1	26.5 ± 5.5	0.785	23.3 ± 3.2	23.2 ± 3.3	0.714
HbA_{1c}, mmol/mol	60.8 ± 14.7	61.9 ± 16.5	0.742	81.4 ± 12.7	81.2 ± 13.2	0.878	53.4 ± 9.5	52.5 ± 11.0	0.523	53.6 ± 9.9	52.5 ± 9.4	0.213	54.0 ± 8.0	53.9 ± 8.4	0.834
Current or ex-smoking, %	20.8	39.3	0.108	40.7	41.5	0.999	67.1	47.3	0.002	52.6	44.3	0.095	51.5	38.9	0.005
Hypertension, %	66.7	38.6	0.011	78.0	64.7	0.050	80.6	71.5	0.137	78.9	59.1	<0.001	77.2	66.5	0.012
Dyslipidemia, %	66.7	55.5	0.385	89.8	82.4	0.193	97.0	87.3	0.029	77.2	77.4	0.999	76.5	76.2	0.999
eGFR, mL/min per1.73 m^2	72.0 ± 9.5	79.7 ± 25.2	0.001	73.7 ± 12.3	77.5 ± 26.9	0.032	70.3 ± 11.5	69.1 ± 29.0	0.439	72.2 ± 12.8	78.7 ± 28.0	<0.001	71.8 ± 11.6	67.9 ± 19.9	<0.001

Proteinuria (Dataset C)

	SAID			SIDD			SIRD			MOD			MARD		
	Proteinuria			Proteinuria			Proteinuria			Proteinuria			Proteinuria		
Variables	Event	No event	p value	Event	No event	p value	Event	No event	p value	Event	No event	p value	Event	No event	p value
Age, years	56 ± 15	55 ± 15	0.971	63 ± 13	62 ± 13	0.398	61 ± 14.0	59 ± 14	0.285	57 ± 13	55 ± 13	0.035	72 ± 7.6	71 ± 3.6	<0.001
Women, %	54.1	55.8	0.891	47.3	36.5	0.406	63.4	60.4	0.053	36.8	63.2	0.605	62.7	51.5	<0.001
Diabetes duration, years	10.2 ± 9.3	9.7 ± 9.3	0.676	16.3 ± 11.3	13.0 ± 10.7	<0.001	10.1 ± 9.1	8.6 ± 2.2	0.033	16.6 ± 11.9	14.7 ± 11.7	0.005	11.2 ± 7.8	10.1 ± 7.6	0.009
BMI, kg/m^2	23.3 ± 4.7	23.1 ± 4.1	0.693	26.1 ± 5.4	25.8 ± 4.8	0.425	29.5 ± 5.9	28.7 ± 6.5	0.105	26.9 ± 5.3	26.2 ± 5.2	0.021	23.7 ± 3.4	23.1 ± 3.1	<0.001
HbA_{1c}, mmol/mol	62.3 ± 15.7	62.2 ± 16.4	0.958	79.8 ± 11.5	80.4 ± 13.3	0.626	53.0 ± 10.7	51.4 ± 10.4	0.006	53.1 ± 8.9	51.8 ± 9.4	0.012	54.0 ± 8.0	53.4 ± 8.1	0.250
Current or ex-smoking , %	40.5	35.0	0.456	39.9	42.0	0.710	49.2	47.7	0.768	48.3	42.1	0.035	44.8	37.4	0.007
Hypertension, %	45.9	36.4	0.169	67.6	63.5	0.406	81.3	67.3	<0.001	67.4	57.1	<0.001	69.2	65.6	0.199
Dyslipidemia, %	40.5	35.0	0.738	87.2	82.0	0.167	90.2	87.7	0.429	79.9	77.5	0.339	76.1	77.9	0.490
eGFR, mL/min per 1.73 m^2	77.8 ± 25.5	79.8 ± 24.4	0.467	77.9 ± 26.1	77.1 ± 26.5	0.688	69.9 ± 23.8	68.2 ± 27.5	0.298	79.5 ± 26.4	78.1 ± 27.4	0.288	67.5 ± 20.2	68.3 ± 19.4	0.374

Values are presented as mean ± SD, or n (%). P values were obtained by one-way ANOVA test or Chi-square test. SAID: severe autoimmune diabetes; SIDD: severe insulin-deficient diabetes; SIRD: severe insulin-resistant diabetes; MOD: mild obesity-related diabetes; MARD: mild age-related diabetes; BMI: body mass index; HbA_{1c}: hemoglobin A_{1c}; eGFR: estimated glomerular filtration rate; CKD: chronic kidney disease.

ESM Table 8. P values for interaction between diabetes subtypes and risk factors in Cox proportional hazard raito (Model 5) for eGFR and proteinuria events

CKD stage G3b

	Age (years)			Sex			Diabetes duration (years)				BMI (kg/m^2)				
	< 65	65 ≤	P for interaction	Men	Women	P for interaction	< 2	2-9	≥ 10	P for interaction	< 18.5	18.4 - 21.9	22.0 - 24.9	25 ≤	P for interaction
Variables	HR (95%CI)	HR (95%CI)		HR (95%CI)	HR (95%CI)		HR (95%CI)	HR (95%CI)	HR (95%CI)		HR (95%CI)	HR (95%CI)	HR (95%CI)	HR (95%CI)	
SAID	0.35 (0.16 - 0.75)	1.26 (0.85 - 1.86)	0.643	0.92 (0.55 - 1.53)	1.15 (0.73 - 1.85)	0.643	1.53 (0.42 - 5.54)	0.87 (0.44 - 1.74)	0.99 (0.65 - 1.52)	0.643	0.63 (0.34 - 1.21)	1.08 (0.55 - 2.12)	1.16 (0.65 - 2.06)	1.02 (0.58 - 1.81)	0.643
SIDD	0.91 (0.47 - 1.76)	1.32(0.99 - 1.77)	0.381	1.35 (0.95 - 1.90)	1.49 (0.98 - 2.25)	0.381	1.47 (0.40 - 5.38)	1.63 (0.75 - 3.55)	1.18 (0.88 - 1.58)	0.381	1.00 (0.54 - 1.84)	1.32 (0.67 - 2.61)	1.30 (0.82 - 2.06)	1.86 (1.25 - 2.88)	0.381
SIRD	0.64 (0.39 - 1/05)	1.31 (1.08 - 1.75)	0.828	1.24 (1.00 - 1.53)	1.23 (0.92 - 1.64)	0.828	1.91 (0.96 - 3.80)	1.55 (1.11 - 2.13)	1.06 (0.85 - 1.31)	0.828	1.92 (1.05 - 3.54)	1.02 (0.67 - 1.55)	1.37 (0.99 - 1.90)	1.44 (1.11 - 1.88)	0.828
MOD	0.56 (0.35 - 0.91)	1.45 (1.14 - 1.84)	0.029	1.24 (0.97 - 1.59)	1.42 (0.99 - 2.03)	0.029	0.78 (0.15 - 4.03)	1.24 (0.73 - 2.11)	1.15 (0.92 - 1.44)	0.029	0.93 (0.57 - 1.52)	0.99 (0.61 - 1.58)	1.18 (0.80 - 1.74)	1.46 (1.08 - 1.97)	0.029
MARD	1.00 (ref)	1.00 (ref)	–	1.00 (ref)	1.00 (ref)	–	1.00 (ref)	1.00 (ref)	1.00 (ref)	–	1.00 (ref)	1.00 (ref)	1.00 (ref)	1.00 (ref)	–

HbA1c (mmol/mol,)			Hypertension			Dyslipidemia			Smoking		
< 63	≥ 63	P for interaction	no	yes	P for interaction	no	yes	P for interaction	never	Current or ex-smoking	P for interaction
HR (95%CI)	HR (95%CI)		HR (95%CI)	HR (95%CI)		HR (95%CI)	HR (95%CI)		HR (95%CI)	HR (95%CI)	
0.95 (0.63 - 1.45)	1.14 (0.62 - 2.07)	0.643	0.92 (0.39 - 2.23)	1.32 (0.76 - 2.30)	0.643	0.75 (0.29 - 1.81)	1.25 (0.72 - 2.18)	0.643	1.85 (1.07 - 3.20)	0.39 (0.15 - 1.02)	0.643
4.04 (0.56 - 29.32)	1.24 (0.85 - 1.80)	0.381	1.19 (0.48 - 2.94)	1.11 (0.68 - 1.81)	0.381	0.63 (0.19 - 2.26)	1.24 (0.77 - 1.99)	0.381	1.63 (0.92 - 2.87)	0.80 (0.41 - 1.56)	0.381
1.21 (1.00 - 1.46)	1.24 (0.83 - 1.84)	0.828	2.23 (1.05 - 4.79)	1.46 (1.00 - 2.12)	0.828	0.28 (0.06 - 1.40)	1.90 (1.33 - 2.71)	0.828	1.64 (0.96 - 2.81)	1.43 (0.91 - 2.23)	0.828
1.18 (0.95 - 1.48)	1.24 (0.79 - 1.96)	0.029	0.96 (0.49 - 1.92)	1.18 (0.81 - 1.71)	0.029	0.80 (0.40 - 1.59)	1.16 (0.80 - 1.67)	0.029	1.49 (0.94 - 2.38)	0.82 (0.52 - 1.29)	0.029
1.00 (ref)	1.00 (ref)	–	1.00 (ref)	1.00 (ref)	–	1.00 (ref)	1.00 (ref)	–	1.00 (ref)	1.00 (ref)	–

Proteinuria

	Age (years)			Sex			Diabetes duration (years)				BMI (kg/m^2)				
	< 65	65 ≤	P for interaction	Men	Women	P for interaction	< 2	2-9	≥ 10	P for interaction	< 18.5	18.4 - 21.9	22.0 - 24.9	25 ≤	P for interaction
Variables	HR (95%CI)	HR (95%CI)		HR (95%CI)	HR (95%CI)		HR (95%CI)	HR (95%CI)	HR (95%CI)		HR (95%CI)	HR (95%CI)	HR (95%CI)	HR (95%CI)	
SAID	0.35 (0.16 - 0.75)	1.26 (0.85 - 1.86)	0.040	0.92 (0.55 - 1.53)	1.15 (0.73 - 1.85)	0.040	1.53 (0.42 - 5.54)	0.87 (0.44 - 1.74)	0.99 (0.65 - 1.52)	0.040	0.63 (0.34 - 1.21)	1.08 (0.55 - 2.12)	1.16 (0.65 - 2.06)	1.02 (0.58 - 1.81)	0.040
SIDD	0.91 (0.47 - 1.76)	1.32(0.99 - 1.77)	0.546	1.35 (0.95 - 1.90)	1.49 (0.98 - 2.25)	0.546	1.47 (0.40 - 5.38)	1.63 (0.75 - 3.55)	1.18 (0.88 - 1.58)	0.546	1.00 (0.54 - 1.84)	1.32 (0.67 - 2.61)	1.30 (0.82 - 2.06)	1.86 (1.25 - 2.88)	0.546
SIRD	0.64 (0.39 - 1/05)	1.31 (1.08 - 1.75)	0.096	1.24 (1.00 - 1.53)	1.23 (0.92 - 1.64)	0.096	1.91 (0.96 - 3.80)	1.55 (1.11 - 2.13)	1.06 (0.85 - 1.31)	0.096	1.92 (1.05 - 3.54)	1.02 (0.67 - 1.55)	1.37 (0.99 - 1.90)	1.44 (1.11 - 1.88)	0.096
MOD	0.56 (0.35 - 0.91)	1.45 (1.14 - 1.84)	0.368	1.24 (0.97 - 1.59)	1.42 (0.99 - 2.03)	0.368	0.78 (0.15 - 4.03)	1.24 (0.73 - 2.11)	1.15 (0.92 - 1.44)	0.368	0.93 (0.57 - 1.52)	0.99 (0.61 - 1.58)	1.18 (0.80 - 1.74)	1.46 (1.08 - 1.97)	0.368
MARD	1.00 (ref)	1.00 (ref)	–	1.00 (ref)	1.00 (ref)	–	1.00 (ref)	1.00 (ref)	1.00 (ref)	–	1.00 (ref)	1.00 (ref)	1.00 (ref)	1.00 (ref)	–

HbA1c(mmol/mol,)			Hypertension			Dyslipidemia			Smoking		
< 63	≥ 63	P for interaction	no	yes	P for interaction	no	yes	P for interaction	never	Current or ex-smoking	P for interaction
HR (95%CI)	HR (95%CI)		HR (95%CI)	HR (95%CI)		HR (95%CI)	HR (95%CI)		HR (95%CI)	HR (95%CI)	
0.95 (0.63 - 1.45)	1.14 (0.62 - 2.07)	0.040	0.54 (0.23 - 1.26)	0.92 (0.64 - 1.34)	0.040	0.30 (0.13 - 0.72)	1.01 (0.70 - 1.47)	0.040	0.58 (0.36 - 0.94)	1.03 (0.63 - 1.68)	0.040
4.04 (0.56 - 29.32)	1.24 (0.85 - 1.80)	0.546	1.89 (0.96 - 3.73)	0.73 (0.56 - 0.96)	0.546	1.10 (0.53 - 2.28)	0.79 (0.61 - 1.02)	0.546	0.68 (0.49 - 0.94)	1.06 (0.73 - 1.55)	0.546
1.21 (1.00 - 1.46)	1.24 (0.83 - 1.84)	0.096	0.80 (0.48 - 1.34)	0.87 (0.72 - 1.04)	0.096	0.73 (0.44 - 1.21)	0.88 (0.73 - 1.05)	0.096	0.84 (0.67 - 1.05)	0.96 (0.73 - 1.25)	0.096
1.18 (0.95 - 1.48)	1.24 (0.79 - 1.96)	0.368	1.00 (0.57 - 1.76)	1.11 (0.90 - 1.37)	0.368	1.40 (0.85 - 2.30)	1.09 (0.88 - 1.35)	0.368	1.00 (0.78 - 1.31)	1.32 (0.97 - 1.80)	0.368
1.00 (ref)	1.00 (ref)	–	1.00 (ref)	1.00 (ref)	–	1.00 (ref)	1.00 (ref)	–	1.00 (ref)	1.00 (ref)	–

SAID: severe autoimmune diabetes; SIDD: severe insulin-deficient diabetes; SIRD: severe insulin-resistant diabetes; MOD: mild obesity-related diabetes; MARD: mild age-related diabetes; BMI: body mass index; HbA1c: Hemoglobin A1c; eGFR: estimated glomerular filtration rate; CKD: chronic kidney disease.

ESM Table 9. Baseline characteristics of diabetes subtypes in Dataset E

Variables	SIDD *n* =656	SIRD *n* =351	MOD *n* =1,178	MARD *n* =1,767	*p* value
Women, %	50.4	32.2	35.6	45.7	<0.001
Age at diagnosis, years	47.5 ± 11.7	50.5 ± 12.6	39.6 ± 8.5	60.9 ± 8.5	<0.001
Smoking, %	51.4	54.2	47.0	50.6	<0.001
Anthropometry					
BMI, kg/m^2	25.9 ± 5.5	29.5 ± 7.1	26.5 ± 5.5	23.0 ± 3.3	<0.001
Waist circumference, cm	95 ± 16	102 ± 17	95 ± 15	88 ± 10	<0.001
Systolic blood pressure, mmHg	129 ± 19	130 ± 18	128 ± 17	129 ± 17	<0.001
Diastolic blood pressure, mmHg	75 ± 12	77 ± 13	75 ±12	72 ± 12	<0.001
Biochemistry					
HbA_{1c}					
%	9.6 ± 1.2	7.1 ± 1.0	7.1 ± 0.9	7.2 ± 0.8	<0.001
mmol/mol	81.2 ± 12.8	54.0 ± 10.8	54.5 ± 9.0	55.4 ±8.3	<0.001
HOMA2-β	46.8 ± 23.1	146.1 ± 62.1	66.1 ± 32.6	62.2 ± 28.2	<0.001
HOMA2-IR	1.76 ± 1.13	4.80 ± 1.79	1.79 ± 0.95	1.76 ± 0.96	<0.001
Fasting plasma glucose					
mg/dL	153 ± 30	133 ± 31	134 ± 29	136 ± 27	<0.001
mmol/l	8.5 ± 1.6	7.4 ± 1.7	7.4 ± 1.6	7.5 ± 1.5	<0.001
eGFR, mL/min per 1.73 m^2	77 ± 29	65 ± 27	75 ± 26	67 ± 19	<0.001

Values are presented as mean ± SD, or n (%). P values were obtained by one-way ANOVA or Chi-square test. SIDD: severe insulin-deficient diabetes; SIRD: severe insulin-resistant diabetes; MOD: mild obesity-related diabetes; MARD: mild age-related diabetes; BMI: body mass index; HbA_{1c}: hemoglobin A_{1c}; HOMA2-β: homeostatic model assessment 2 estimates of β-cell function; HOMA2-IR: homeostatic model assessment 2 estimates of insulin resistance; eGFR: estimated glomerular filtration rate.

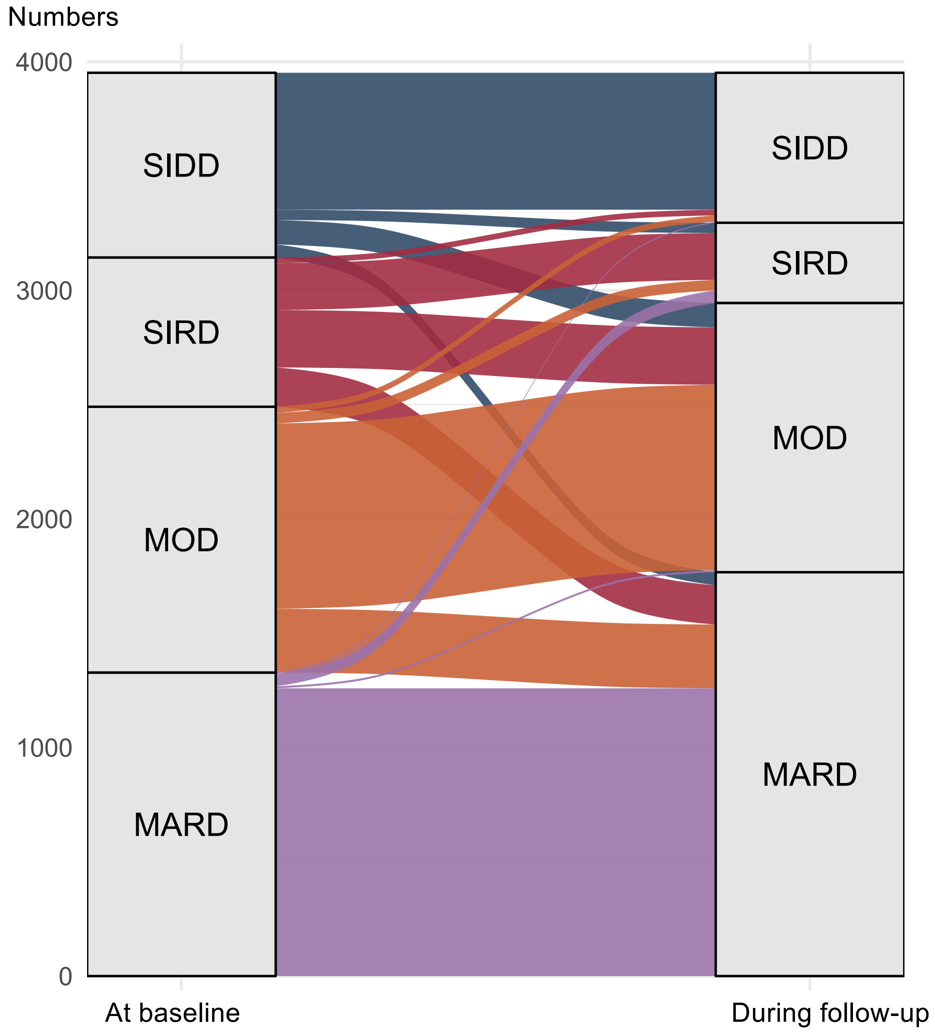

At baseline		During follow-up				
Subtype		Subtype			Stage 3b	
	n		*n*	%	Event/numbers	%
SIDD	808	SIDD	599	74.1	37/392	9.4
		SIRD	45	5.6	5/32	15.6
		MOD	107	13.2	5/75	6.7
		MARD	57	7.1	5/39	12.8
SIRD	653	SIDD	25	3.8	2/11	18.2
		SIRD	205	31.4	18/107	16.8
		MOD	251	38.4	16/155	10.3
		MARD	172	26.3	8/82	9.8
MOD	1,163	SIDD	27	2.3	4/18	22.2
		SIRD	46	4.0	3/28	10.7
		MOD	811	69.7	43/556	7.7
		MARD	279	24.0	18/185	9.7
MARD	1,328	SIDD	5	0.4	0/3	0.0
		SIRD	55	4.1	3/32	9.4
		MOD	9	0.7	0/3	0.0
		MARD	1,259	94.8	73/789	9.1

ESM Fig.8 Sankey diagram showing reproducibility pattern of diabetes subtypes. Dataset E (*n*=3,952) was extracted from Dataset B for cluster reproducibility analysis. This analysis included participants who had Ahlqvist's variables at both the baseline and follow-up assessments (mean interval: 5.0 ± 1.2 years). Right panel shows numbers and % transitioned to other subtypes or % maintained original subtypes.

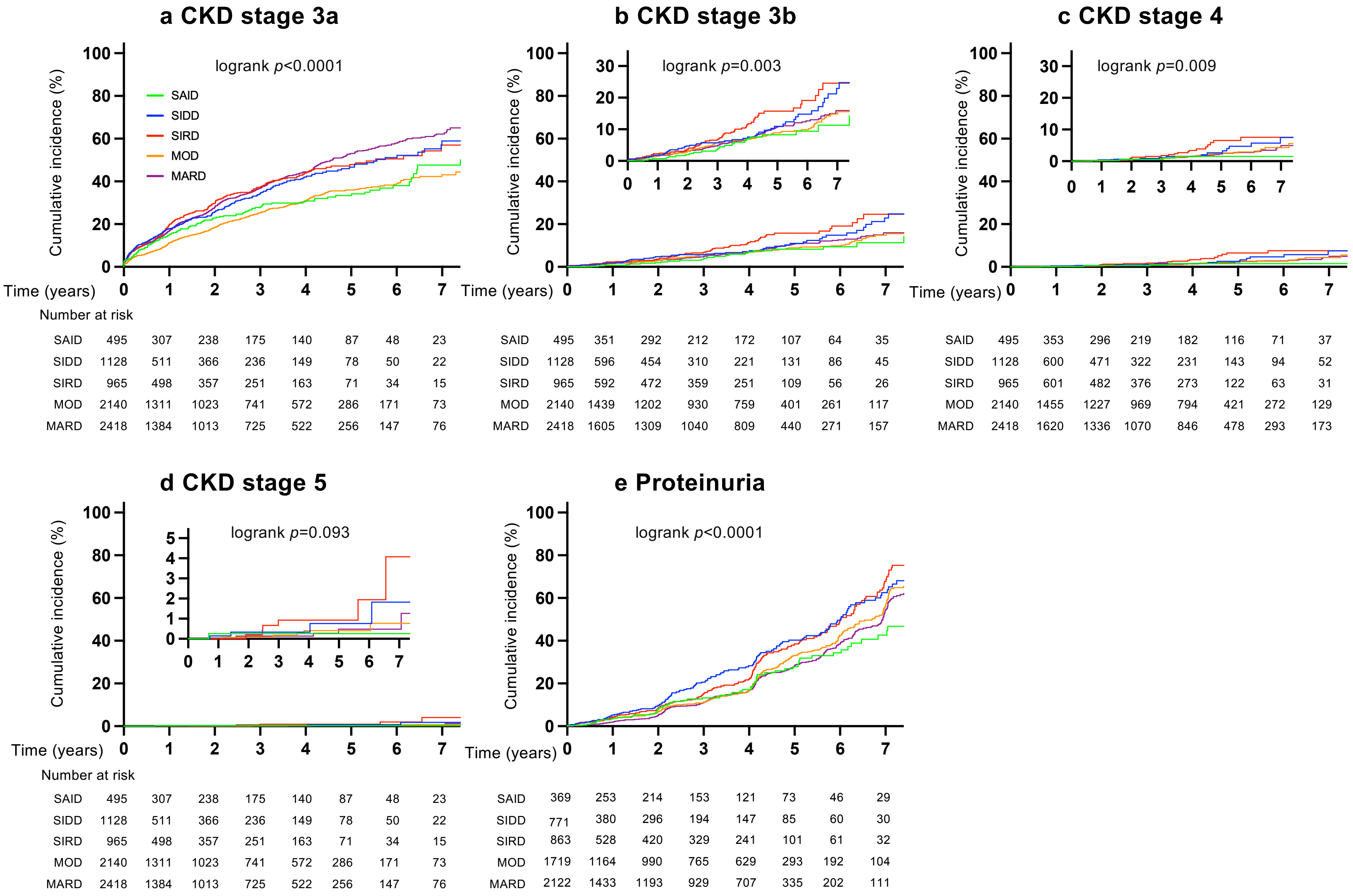

ESM Fig.9 Seven-years censored Kaplan-Meier curves for the development of (**a**) stage 3a (eGFR <60 mL/min/1.73 m^2), (**b**) stage 3 (eGFR <45 mL/min/1.73 m^2), (**c**) stage 4 (eGFR <30 mL/min/1.73 m^2), (**d**) stage 5 (eGFR <15 mL/min/1.73 m^2), (**e**) proteinuria in individuals with SAID (green lines), SIDD (blue lines, severe insulin-deficient diabetes), SIRD (red lines, severe insulin-resistant diabetes), MOD (orange lines, mild obesity-related diabetes), and MARD (purple lines, mild age-related diabetes). eGFR: estimated glomerular filtration rate. Analyses for stages 3a, 3b, 4, and 5 were performed using Dataset B n=7,146) and those for proteinuria using Dataset C (*n*=5,844) in a Japanese diabetes cohort database (J-DREAMS registry). Logrank p values are shown.
